# High Temperature Drives Topoisomerase Mediated Chromosomal Break Repair Pathway Choice

**DOI:** 10.3390/cancers13102315

**Published:** 2021-05-12

**Authors:** Mohamed E. Ashour, Walaa Allam, Waheba Elsayed, Reham Atteya, Menattallah Elserafy, Sameh Magdeldin, Mohamed K. Hassan, Sherif F. El-Khamisy

**Affiliations:** 1 Center for Genomics, Helmy Institute for Medical Science, Zewail City of Science and Technology, Giza 12578, Egypt; ashour@wustl.edu (M.E.A.); walaaramadan@gmail.com (W.A.); p-walsayed@zewailcity.edu.eg (W.E.); p-rattaeya@zewailcity.edu.eg (R.A.); melserafy@zewailcity.edu.eg (M.E.); 2 Proteomics and Metabolomics Research Program, Children Cancer Hospital (CCHE 57357), Cairo 11441, Egypt; Sameh.Magdeldin@57357.org; 3 Physiology Department, Faculty of Veterinary Medicine, Suez Canal University, Ismailia 41522, Egypt; 4 Biotechnology Program, Biology Department, Faculty of Science, Port Said University, Port Said 42522, Egypt; 5 The Healthy Lifespan and the Neuroscience Institutes, University of Sheffield, South Yorkshire, Sheffield S10 2TN, UK; 6 The Institute of Cancer Therapeutics, University of Bradford, West Yorkshire BD7 1DP, UK

**Keywords:** topoisomerase, TDP1, TDP2, cancer, ageing, hyperthermia, genomic instability

## Abstract

**Simple Summary:**

Targeting topoisomerases has been widely used as anticancer therapeutics. Exposure to high temperature (hyperthermia) protects cells from the cytotoxic effect of topoisomerase-targeting therapeutics, yet the mechanism remains unknown. Here, we report that hyperthermia inhibits the nucleolytic processing of topoisomerase-induced DNA damage and drives repair to a more faithful pathway mediated by TDP1 and TDP2. We further show that hyperthermia suppresses topoisomerase-induced chromosomal translocation and hallmarks of inflammation, which has broad implications in cancer development and therapy.

**Abstract:**

Cancer-causing mutations often arise from inappropriate DNA repair, yet acute exposure to DNA damage is widely used to treat cancer. The challenge remains in how to specifically induce excessive DNA damage in cancer cells while minimizing the undesirable effects of genomic instability in noncancerous cells. One approach is the acute exposure to hyperthermia, which suppresses DNA repair and synergizes with radiotherapy and chemotherapy. An exception, however, is the protective effect of hyperthermia on topoisomerase targeting therapeutics. The molecular explanation for this conundrum remains unclear. Here, we show that hyperthermia suppresses the level of topoisomerase mediated single- and double-strand breaks induced by exposure to topoisomerase poisons. We further uncover that, hyperthermia suppresses hallmarks of genomic instability induced by topoisomerase targeting therapeutics by inhibiting nuclease activities, thereby channeling repair to error-free pathways driven by tyrosyl-DNA phosphodiesterases. These findings provide an explanation for the protective effect of hyperthermia from topoisomerase-induced DNA damage and may help to explain the inverse relationship between cancer incidence and temperature. They also pave the way for the use of controlled heat as a therapeutic adjunct to topoisomerase targeting therapeutics.

## 1. Introduction

Cancer is a leading cause of mortality with variable incidence among populations [1]. Analysis of epidemiological data has uncovered an inverse pattern of geographical cancer distribution and annual temperature. Countries with low average annual temperatures exhibit the highest incidence of cancer [1,2,3,4]. The two populations living in the coldest environments, Alaska Indians and Inuit, exhibit high cancer incidence [3,5]. Although human thermoregulation systems sustain body core temperature at 37 °C, exposure to extreme or sustained temperatures overwhelms the body’s thermoregulatory capacity especially at old age, leading to hypothermia (<35 °C) or hyperthermia (>40.5 °C) [6,7]. How the changes in environmental and body temperature affect cancer development remains unclear.

Hyperthermia treatment, also known as thermotherapy, is an ancient treatment used to treat cancer, dating back to 5000 BC [8]. Thermotherapy is applied by regionally heating the tumor to 40–45 °C [9,10]. Hyperthermia induces a wide range of cellular effects, including mitotic dysfunction, cytoskeletal damage, alternations in membrane structure, metabolic dysfunction, DNA damage, interference with cell cycle, and protein denaturation [9,11,12]. While noncancerous cells can tolerate temperature up to 42–45 °C, cancerous cells are more susceptible [13,14,15]. Hyperthermia enhances the effectiveness of radiotherapy and chemotherapy, including cisplatin, carboplatin, cyclophosphamide, ifosfamide, melphalan, mitomycin C, and histone deacetylase inhibitors [10,16,17,18,19]. A well-studied example is the synergistic effect of hyperthermia with radiation therapy (thermo-radiosensitization) [20]. Thermo-radiosensitization was shown to be due to increased tumor oxygenation uptake through enhancing blood flow, which overcomes radiation resistance to hypoxia [10]. Hyperthermia has also been shown to increase radiation-induced double-strand breaks (DSBs) [21,22], through heat-mediated inactivation of the DSB repair pathways, non-homologous end joining (NHEJ) and homologous recombination (HR) [19,23,24,25,26].

Hyperthermia inhibits several DNA repair pathways, including components of the DNA base excision repair (BER), nucleotide excision repair (NER), mismatch repair (MMR), single-strand breaks (SSBs) repair, and DSB repair pathways [19,27]. Moreover, hyperthermia induces DNA damage directly, whereby increased levels of 8-oxoguanine, apurinic sites, and deaminated cytosines were identified following heat exposure [9,27,28,29]. While hyperthermia increases DSB formation, this effect was explained as an indirect consequence of interfering with DNA repair mechanisms [9,27,28,30]. Hyperthermia has also been shown to inhibit HR by triggering BRCA2-degradation [23,25,26], which opened new avenues for combination therapies with PARP inhibitors, specifically targeting HR-deficient tumors [31,32].

In contrast to radiation and several chemotherapeutic agents, hyperthermia failed to sensitize cells to topoisomerase (TOP) inhibitors and instead exerted a protective effect. This paradigm remains unexplained [33,34,35,36]. Topoisomerases remove topological stress by cleaving and resealing DNA strands. This mode of catalysis can be dangerous if topoisomerases become covalently linked to DNA, generating an intermediate known as topoisomerase cleavage complex (TOPcc). While TOPcc is reversible, it can be trapped by topoisomerase poisons such as camptothecin and etoposide, targeting TOP1 and TOP2, respectively, and becomes an irreversible DNA–protein crosslink (DPC). DPC interferes with replication and transcription, leading to genome instability or cell death [37,38,39]. Accumulation of TOP-DNA breaks is counteracted by repair factors that precisely disjoin the covalently linked topoisomerases from DNA through the activity of tyrosyl-DNA phosphodiesterases (TDP1 and TDP2), or nucleolytically cleave the DNA, releasing the topoisomerases and a fragment of DNA [37,38,39,40]. The cell’s decision to choose TDPs or nucleases has important consequences on genome stability as TDPs spare the genome from error-prone nucleolytic cleavage [37,38,39,40,41,42,43]. Notably, mutations in genes encoding the TDP proteins lead to neurodegenerative conditions [44,45,46]. Several nucleases are implicated in DPC repair, such as CtIP and MRE11, which participate in processing TOP1cc and TOP2cc, while the structure-specific nucleases, MUS81–EME1 and XPF–ERCC1, are primarily implicated in processing TOP1cc [37,38,39].

The inappropriate error-prone repair of topoisomerase-induced DNA breaks has been associated with cancer, neurodegenerative disease, and autoimmune syndromes [37,47,48,49,50]. The association between topoisomerases and cancer is best illustrated by the role of TOP2cc in inducing chromosomal translocations. This association was first established by the observation that cancer-targeting TOP2 inhibitors develop secondary leukemia by inducing *MLL* and *PML-RARA* translocation [37,49,51]. Moreover, androgen signaling co-recruits androgen receptor and TOP2β to TMPRSS2 and ERG genes. The recruited TOP2β induces de novo TMPRSS2-ERG fusion, resulting in prostate cancer development [52]. Chromosome loop anchors, bound by CTCF and cohesion, were shown to be vulnerable to DSBs mediated by TOP2β, leading to chromosomal rearrangements. The kinetics of TOP2β-mediated translocation can be predicted by cohesin and transcription levels at particular sites [43,53]. Another report has shown that damaged introns with paused RNA pol II, TOP2β, and XRCC4 are enriched in translocation breakpoints [54]. Consistently, the TOP2 inhibitor, etoposide, induces high levels of chromosomal translocations in cells deficient for the TOP2-DNA repair enzyme, TDP2 [41,42]. Translocations that arise in the absence of TDP2 are most likely mediated by a mutagenic DSB repair mechanism that employs endonucleases such as MRE11 [41,42,49,55]. Another link was established between TOPcc and cancer, where TOP1 was shown to mediate a mutagenic pathway to remove ribose contamination from DNA. This unfaithful role has been implicated in 5 bp deletions in highly transcribed genes and in generating lesions that trap PARP1, leading to cell killing [56,57,58].

Although the protective effect of hyperthermia on topoisomerase targeting therapeutics has been reported, the underlying molecular mechanism remains unclear. Moreover, the impact of hyperthermia on topoisomerase-induced genomic instability is unknown. Here, we report that hyperthermia suppresses the level of topoisomerase mediated single- and double-strand breaks induced by exposure to topoisomerase poisons. Furthermore, we uncover that hyperthermia suppresses the level of genomic instability induced by topoisomerase poisons by inhibiting nuclease activities, thereby channeling repair to the error-free TDP pathways. These findings identify a novel mechanism for the protective effect of hyperthermia from topoisomerase-induced genomic instability and could help in understanding the inverse relationship between cancer and environmental temperature.

## 2. Results

### 2.1. Hyperthermia Reduces the Catalytic Activity of TDP1 and TDP2

**Figure 1:**
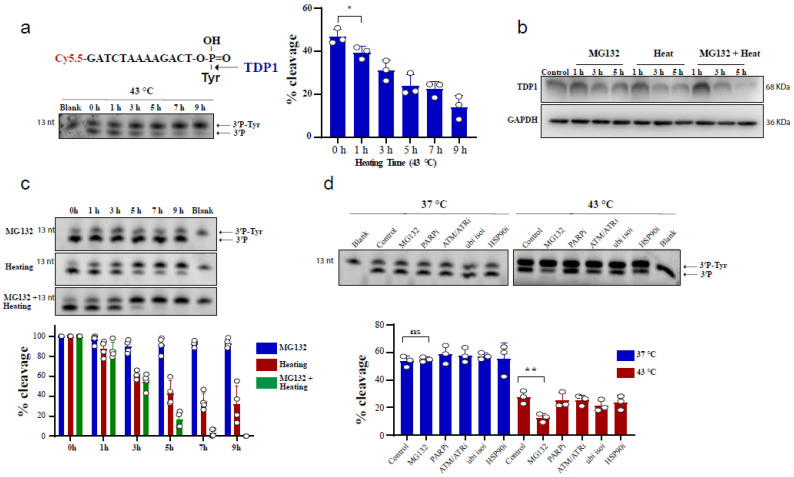
Hyperthermia reduces TDP1 activity. (**a**) RKO cells were incubated for the indicated time at 43 °C. Then, the cells were collected and lysed. Whole cell lysates (0.2 µg) were incubated for 30 min with TDP1 substrate to monitor TDP1 catalytic activity in buffer containing EDTA. Reaction products were separated by 20% denaturing PAGE. TDP1 catalytic activity was quantified as % cleavage of 3′P-Tyr to 3′P. (**b**) TDP1 protein level was determined by Western blot after incubating the RKO cells with 10 µM MG132 and/or heating at 43 °C for the indicated time. (**c**) RKO cells were treated with 10 µM MG132 and/or heating at 43 °C for the indicated time points. TDP1 activity was assessed as in (**a**). TDP1 activities were normalized to control (0 h) and presented as relative percentage change. (**d**) RKO cells were treated with DMSO (Control), 10 µM proteasomei (MG132), 15 µM PARPi (olaparib), 10 µM ATM/ATRi (CGK733), 5 µM ubiquitin isopeptidasei I (ubi isoi; G5) or 1.2 µM HSP90i (17-AAG) at 37 °C (left) or at 43 °C (right) for 3 h. TDP1 activity was assessed as in (**a**). Data represent the mean of at least three biological replicates ± SD. Asterisks denote statistical significance (* *p* < 0.05, and ** *p* < 0.01 as per Student’s *t*-test).

To test the effect of heating (hyperthermia) on TDP1 catalytic activity, we used an in vitro biochemical assay employing a single-stranded oligonucleotide substrate containing a 3′-phosphotyrosine (3′P-tyr) and 5′-fluorophore. The cleaved tyrosine from the substrate leads to faster migration, resulting in a slightly lower molecular weight band, indicative of TDP1 catalytic activity. RKO cells were exposed to 43 °C and whole-cell lysates were incubated with the TDP1 substrate. Exposure to hyperthermia led to a reduction in TDP1 catalytic activity, which was significant following 1 h exposure to heat (Figure 1a). We also observed that increasing heat exposure time led to a time-dependent reduction in TDP1 activity. The reduced activity was associated with a corresponding reduction in TDP1 protein levels (Figure 1b). This effect was not cell-type specific as a similar result was observed in MCF-7 cells (Supplementary Figure S1a) and remained apparent after recovery from heat exposure up to 12 h (Supplementary Figure S1b). Notably, inhibiting the proteasome by MG132 treatment exacerbated the inhibitory effect of hyperthermia on TDP1 catalytic activity and the reduction in TDP1 protein level (Figure 1b,c). This effect was not due to an impact of MG132 on TDP1 transcript levels (Supplementary Figure S1c). It was specific to MG132 as inhibitors for PARP1, ATM/ATR, ubiquitin isopeptidases, and HSP90 did not result in a similar effect (Figure 1d). The synergistic effect of MG132 on hyperthermia-induced suppression of TDP1 activity is in contrast to the rescue of hyperthermia-induced proteasome degradation reported for other DNA repair proteins such as BRCA2 [23].

We recently reported that TDP1 protein levels are regulated by ubiquitination and identified the deubiquitinase (DUB), UCHL3, as the DUB regulating TDP1 proteostasis [59]. Therefore, we considered the possibility that hyperthermia reduces UCHL3 levels, which would increase TDP1 ubiquitination and consequently reduce TDP1 levels. Hyperthermia exposure led to a reduction in UCHL3 protein levels (Supplementary Figure S1d), which could partly explain the inhibitory effect of hyperthermia on TDP1 level and activity. However, this was not sufficient to explain the synergistic effect of hyperthermia and proteasome inhibition on TDP1, as MG132 treatment did not further reduce the hyperthermia-induced reduction in UCHL3 levels (Supplementary Figure S1e). In an attempt to identify hyperthermia dependent factors that regulate TDP1 levels, we compared TDP1 interactome with and without exposure to heat stress using mass spectrophotometry. These experiments identified USP10 as a putative hyperthermia dependent TDP1 partner (Supplementary Table S1). However, depletion of USP10 did not impact TDP1 activity following exposure to hyperthermia (Supplementary Figure S1f,g). A similar approach was also unable to identify putative proteins responsible for BRCA2 degradation following hyperthermia [23].

**Figure 2:**
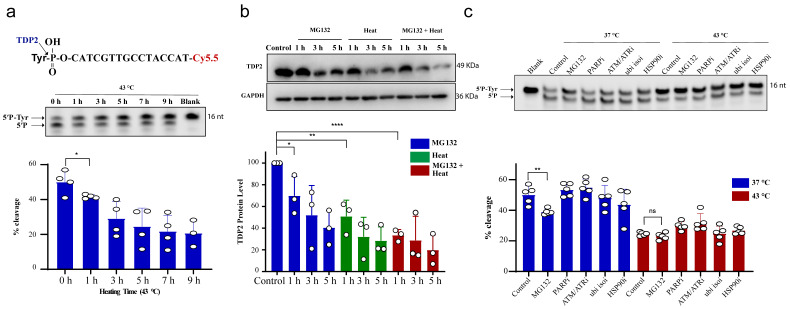
Hyperthermia reduces TDP2 activity. (**a**) RKO cells were incubated for the indicated time at 43 °C. Then, the cells were collected and lysed. Whole cell lysates (15 µg) were incubated with TDP2 substrate for 30 min to monitor TDP2 catalytic activity. Reaction products were separated by 20% denaturing PAGE. TDP2 catalytic activity was quantified as % cleavage of 5′P-Tyr to 5′P. (**b**) TDP2 protein level was determined by Western blot after incubating the RKO cells with 10 µM MG132 and/or heating at 43 °C for the indicated time. TDP2 protein levels were normalized to control and presented as a relative percentage change. (**c**) RKO cells were treated with DMSO (control), 10 µM proteasomal inhibitor (MG132), 15 µM PARPi (olaparib), 10 µM ATM/ATRi (CGK733), 5 µM ubiquitin isopeptidasei I (ubi isoi; G5) or 1.2 µM HSP90i (17-AAG) at 37 °C or 43 °C for 3 h. TDP2 activity was assessed as in (**a**). Data represent the mean of at least three biological replicates ± STD. Asterisks denote statistical significance (* *p* < 0.05, ** *p* < 0.01 and **** *p* < 0.0001 as per Student’s *t*-test).

We next examined TDP2 activity using in vitro biochemical assays employing double-stranded oligonucleotide substrate, forming 4 nucleotide overhang similar to TOP2 induced break and contains a 5′-phosphotyrosine (5′P-tyr) and 3′-fluorophore. Similar to TDP1, exposure of RKO cells to 43 °C led to a reduction in TDP2 catalytic activity, which was significant following 1 h exposure to heat (Figure 2a). We also observed that increasing heat exposure time led to a time-dependent reduction in TDP2 activity. This was also concomitant with a reduction in TDP2 protein level (Figure 2b). MG132 treatment reduced TDP2 activity as hyperthermia (Figure 2c), without a corresponding reduction in TDP2 transcript levels (Supplementary Figure S1c). Taken together, we conclude that hyperthermia reduces the level and activity of TDP1 and TDP2.

### 2.2. Hyperthermia Protects from Topoisomerase-Induced DNA Damage

**Figure 3:**
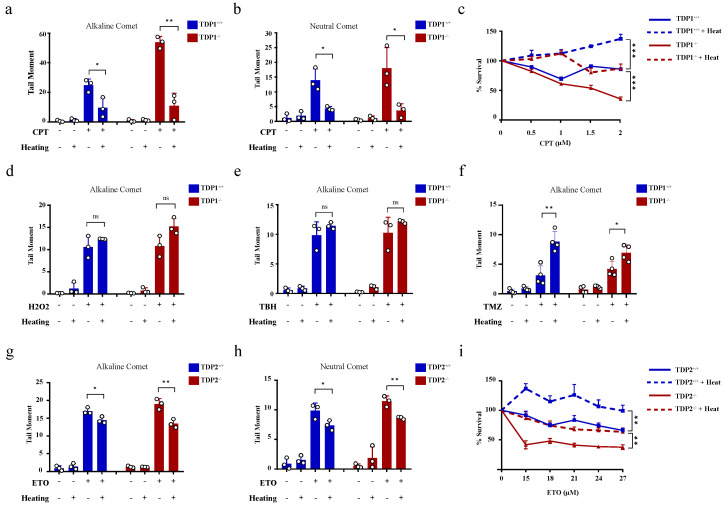
Hyperthermia reduces the level of topoisomerase-induced DNA damage through a TDP independent mechanism. (**a**,**b**) G1 synchronized TK6^TDP1+/+^ and TK6^TDP1-/-^ cells were incubated at 37 or 43 °C for 3 h, then 100 µM CPT was added to the cells for 45 min. DNA breaks, including single and double strand breaks, were quantified by alkaline comet assay (**a**), whereas DNA double strand breaks were quantified by the neutral comet assays (**b**). (**c**) Short term viability assay was used to determine the effect of hyperthermia on CPT-induced cytotoxicity. TK6^TDP1+/+^ and TK6^TDP1-/-^ were incubated at 37 or 43 °C for 1 h. Then, CPT was added at the indicated concentration for 1 h, followed by washing the cells with PBS and keeping the cells in fresh media for 3 days. CellTiter-Blue was used to measure cell viability. (**d**,**e**) G1 synchronized TK6^TDP1+/+^ and TK6^TDP1-/-^ cells were incubated at 37 or 43 °C for 3 h, then 80 µM hydrogen peroxide (H_2_O_2_) (**d**) or 0.075% tertiary-butylhydroperoxide (TBH) (**e**) was added to the cells for 30 min. DNA strand breaks were quantified by the alkaline comet assays. (**f**) G1 synchronized TK6^TDP1+/+^ and TK6^TDP1-/-^ cells were incubated at 37 or 43 °C for 3 h, then 120 µM Temozolomide (TMZ) was added to the cells for 12 h. DNA strand breaks were quantified by the alkaline comet assays. (**g**,**h**) G1 synchronized MEF^TDP2+/+^ and MEF^TDP2-/-^ cells were incubated at 37 or 43 °C for 3 h, then 100 µM etoposide (ETO) was added for 2 h. DNA strand breaks were quantified by the alkaline comet assays (**g**) or neutral comet (**h**). (**i**) Short term viability assay was used to determine the effect of hyperthermia on ETO-induced cytotoxicity. MEF^TDP2+/+^ and MEF^TDP2-/-^ were incubated at 37 or 43 °C for 1 h. Then, ETO was added at the indicated concentrations for 1 h, followed by washing the cells with PBS, and keeping the cells in fresh media for 5 days. CellTiter-Blue was used to measure cell viability. Data are the average of at least three biological replicates ± SD. Asterisks denote statistical significance (* *p* < 0.05, ** *p* < 0.01 and *** *p* < 0.001; Student’s *t*-test).

The hyperthermia-induced reduction in TDP1 and TDP2 level and activities led to the proposition that hyperthermia could consequently increase topoisomerase-induced DNA damage. To examine the effect of hyperthermia on topoisomerase-induced DNA damage, we used TOP poisons, which result in TOP-DNA breaks. To eliminate the potential effect of hyperthermia on cell cycle, we synchronized wild-type (TK6^TDP1+/+^) and TDP1 knockout (TK6^TDP1-/-^) cells in the G1 by double thymidine block and then treated cells with the TOP1 poison, camptothecin (CPT) and quantified DNA strand breaks by the comet assay. In contrast to our predictions, pre-exposure of cells to 43 °C suppressed CPT-induced single-strand breaks (SSBs) and double-strand breaks (DSBs), as measured by the comet assays (Figure 3a,b). The alkaline comet assays measure both SSBs and DSBs, but primarily SSBs because of their increased abundance relative to DSBs, whereas the neutral comet assays measure DSBs [44,60]. We noted that, the absence of TDP1 did not affect the suppression of CPT induced breaks by exposure to hyperthermia. Consistently, hyperthermia antagonized the cytotoxic effect of CPT in both TK6^TDP1+/+^ and TK6^TDP1-/-^ cells, as measured by viability assays (Figure 3c). Notably, our viability data and the hyperthermia conditions used here are consistent with the published reports showing that hyperthermia protects cells from the cytotoxic effect of topoisomerase inhibitors [33,34,35,36]. The possibility that hyperthermia reduced the formation of TOPcc was excluded since multiple independent reports have shown that hyperthermia did not reduce the activity of TOP1, TOP2 or the level of TOP1cc and TOP2cc [33,35,36]. On the contrary, it has been suggested that hyperthermia induces the formation of stable TOP1cc during early S-phase [61]. Consistent with the published literature, we also did not observe a detectable reduction in TOP1 activity following hyperthermia (Supplementary Figure S2a).

We next considered the possibility that heat shock proteins could mediate the protective effect of hyperthermia. To test this, we quantified DNA strand breaks after exposure to hyperthermia with and without pretreatment with inhibitors for HSP70 (Pifithrin-μ), HSP90 (17-AAG) or the proteasome (MG132). None of these inhibitors reversed the protective effect of hyperthermia on CPT-induced strand breaks (Supplementary Figure S2b–d). We then wondered whether such a protective effect of hyperthermia applies to other SSB-inducing agents. To test this, we used hydrogen peroxide and tert-butyl hydroperoxide (TBH), which are established inducers of oxidative SSBs. Unlike CPT, hyperthermia did not protect cells from hydrogen peroxide and TBH-induced SSBs (Figure 3d,e). Furthermore, hyperthermia enhanced the level of DNA damage induced by the alkylating agent, temozolomide (Figure 3f). These results suggest that the protective effect of hyperthermia is specific to topoisomerase poisons. To test if this is also the case for TOP2 mediated DNA damage, we synchronized wild-type (MEF^TDP2+/+^) and TDP2 knockout (MEF^TDP2-/-^) mouse embryonic fibroblasts in G1 and exposed cells to the TOP2 inhibitor, etoposide. Pre-exposure to 43 °C also suppressed etoposide-induced SSBs and DSBs in both cell lines (Figure 3g,h). Consistently, hyperthermia protected both cell lines from cell death induced by etoposide, as measured by viability assays (Figure 3i). Taken together, we conclude that hyperthermia suppresses topoisomerase-induced DNA damage through a TDP-independent mechanism.

### 2.3. Hyperthermia Favors an Error-Free Repair for Topoisomerase-Induced DNA Damage

**Figure 4:**
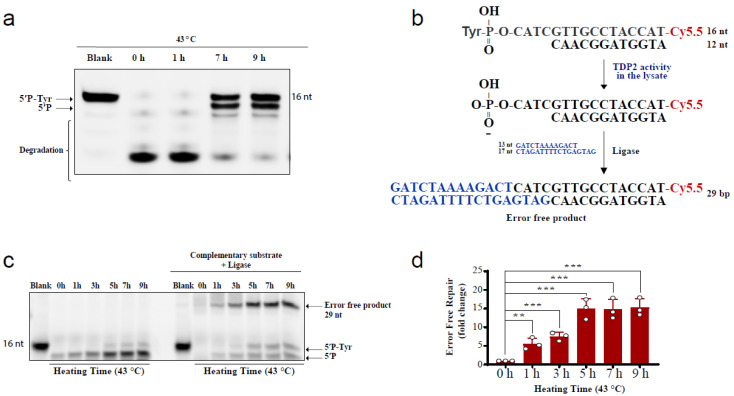
Hyperthermia inhibits nonspecific nucleases and channels the damage to TDP2-mediated error-free repair. (**a**) RKO cells were incubated for the indicated time at 43 °C. Then, cells were collected and lysed. Whole cell lysates (15 µg) were incubated with TDP2 substrate for 1.5 h to monitor TDP2 catalytic activity. Reaction products were separated by 20% denaturing PAGE. TDP2 catalytic activity was quantified as % cleavage of 5′P-Tyr to 5′P. (**b**) Schematic representation for the in vitro reaction and the substrates that were used in panel (**c**). (**c**) RKO cells were treated with hyperthermia at 43 °C for the indicated time, and then the cells were collected, and lysed. The whole cell lysates were incubated with TDP2 substrate in the presence or absence of complementary substrate, and T4 ligase. (**d**) Quantification of the fold increase in the error-free repair in (**c**). Data are the average of at least three biological replicates ± STD. Asterisks denote statistical significance (** *p* < 0.01 and *** *p* < 0.001; Student’s *t*-test).

The above comet results have shown that hyperthermia decreases the amount of TOP-induced DNA damage in a TDP-independent manner. Yet, the fate of the remaining DNA damage following hyperthermia remains unclear. Trapped topoisomerases can be removed by one of two alternative pathways, TDPs or nucleases. The heat-induced reduction in the activity and level of TDPs (Figure 1 and Figure 2) suggests that the nuclease pathway is likely responsible for processing the remaining TOP-induced DNA damage. To test this hypothesis, we examined the effect of heat exposure on nucleases. In the absence of hyperthermia, incubation of the TDP2 substrate with cell lysates for a prolonged 90 min period led to degradation of the 5′P-tyr and 5′P bands. In contrast, prior exposure of cells to 43 °C led to a remarkable protection from degradation (Figure 4a). These results suggest that the nuclease activities are reduced during heat stress. Since TDP2 produces directly ligatable ends that require no further processing [41], we incubated the products of TDP2 reaction with a complementary DNA duplex substrate and T4 DNA ligase to monitor full repair products (Figure 4b,c). Only one hour exposure to 43 °C increased the amount of full repair products ~5-fold, while five hours increased it ~15-fold (Figure 4d). Notably, TDP2^+/+^ and TDP2^-/-^ cells used in this study have been previously employed to show lack of full ligation in the absence of TDP2 because nuclease processing produces nonligatable DNA termini [41]. As expected, supplementing the reaction with exogenous nucleases reversed the protective effect of hyperthermia (Supplementary Figure S3a). The increased formation of full reaction products by heat exposure could either be due to increased TDP2 activity or inhibition of nuclease activities. The former is unlikely as we showed that hyperthermia suppresses TDP2 level and activity (Figure 2a,b). Thus, these observations suggest that although hyperthermia inhibits the activity of both TDP2 and nucleases, the inhibitory effect on nucleases is predominant, thereby favoring a TDP2 error-free repair. Furthermore, these observations show that nucleases contribute to the DNA damage induced following TOP poisoning and that hyperthermia inhibits this nonspecific degradation by suppressing the nuclease activity, which may explain the reduction in the extent of DNA damage quantified in Figure 3.

**Figure 5:**
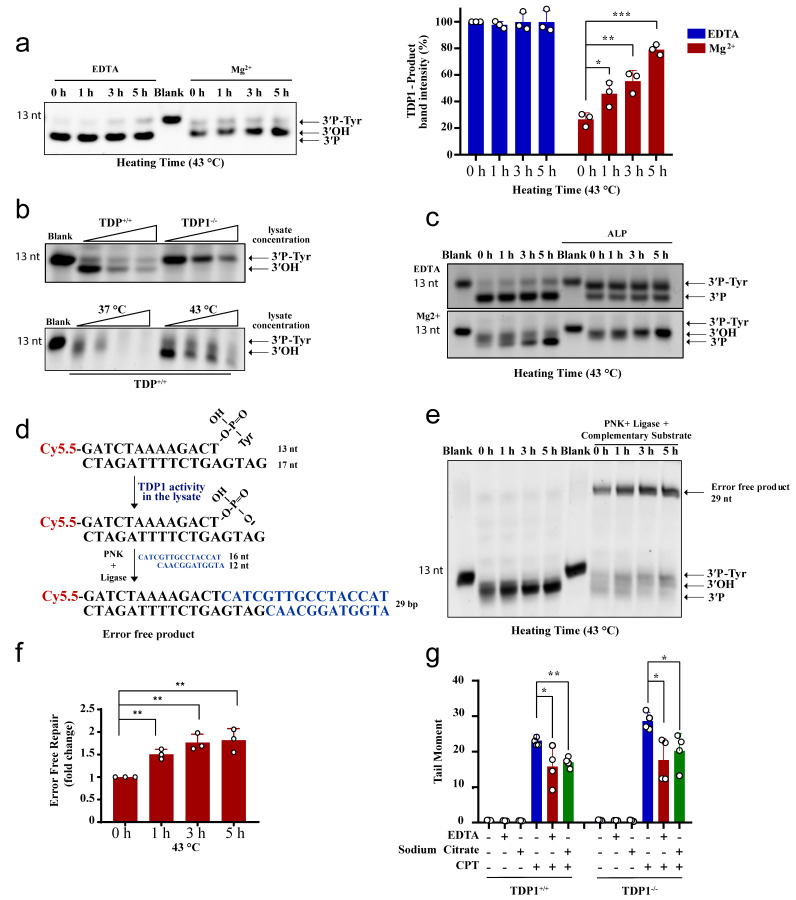
Hyperthermia inhibits nonspecific nucleases and channels the damage to TDP1-mediated error-free repair. (**a**) TK6^TDP1+/+^ cells were treated with hyperthermia at 43 °C for the indicated time. Then, the cells were collected and lysed. Whole cell lysates (2.5 µg) were incubated with TDP1 substrate (presented in Figure 2a) for 1 h to monitor TDP1 catalytic activity in buffer containing EDTA or Mg^2+^. Reaction products were separated by 20% denaturing PAGE. The intensity of TDP1-product bands was normalized to 0 h-EDTA sample and presented as percentage change. (**b**) Increased concentration of cell lysates (2.5, 5, and 7.5 µg) from TK6^TDP1+/+^ and TK6^TDP1-/-^ cells were incubated with TDP1 substrate for 1 h in buffer containing Mg^+2^ to assess TDP1 activity as in (**a**). TK6^TDP1+/+^ cells were incubated at 37 or 43 °C for 5 h. Then, increased concentration of whole cell lysates (2.5, 5, and 7.5 µg) from each group were incubated with TDP1 substrate to assess TDP1 activity as in (**a**). (**c**) TK6^TDP1+/+^ cells were treated with hyperthermia at 43 °C for the indicated time, and then the cells were collected and lysed. The whole cell lysates were incubated with TDP1 substrate in the presence or absence of alkaline phosphatase (ALP). (**d**) Model for the in vitro reaction and substrates that were used in panel (**e**). (**e**) TK6^TDP1+/+^ cells were treated with hyperthermia at 43 °C for the indicated time, and then the cells were collected and lysed. The whole cell lysates were incubated with TDP1 substrate either in the presence or absence of complementary substrate, T4 PNK and T4 DNA ligase. (**f**) Quantification of the fold increase in the error-free repair in (**e**). (**g**) G1 synchronized TK6^TDP1+/+^ and TK6^TDP1-/-^ cells were either treated with 100 µM CPT in normal media, or media supplemented with 5 g/L EDTA or 5 g/L sodium citrate for 45 min. The cells were collected and processed by the neutral comet assays. Mean tail moments were quantified for 100 cells per sample per experiment. Data are the average of at least three biological replicates ± SD. Asterisks denote statistical significance (* *p* < 0.05, ** *p* < 0.01 and *** *p* < 0.001; Student’s *t*-test).

We next examined the effect of hyperthermia on TDP1 substrate degradation. TDP1 activity is metal ion independent, and TDP1 reaction is free from Mg^2+^ but contains the chelating agent, EDTA. The absence of metal ions in the TDP1 reaction renders nucleases and PNKP inactive. To examine nuclease activity, we incubated the TDP1 substrate with TK6 cell lysates and compared reaction products in the presence of EDTA or Mg^2+^. Heat exposure did not impact the products of TDP1 catalytic activity when reactions were performed in the presence of EDTA (Figure 5a, left). In contrast, performing the reactions in the presence of Mg^2+^ led to a reduction in the products of TDP1 activity, which was reversed by heat exposure in a time-dependent manner. Again, only 1 h exposure to hyperthermia led to a significant accumulation of TDP1 products, compared to control cells that were not exposed to hyperthermia (Figure 5a, right). These observations were not cell-type specific as they were also observed with lysates prepared from RKO cells (Supplementary Figure S3b). To test if nucleases degrade the TDP1 substrate (3′-PY) or products (3′-P), we compared lysates from wild-type and TDP1 knockout TK6 cells. Both TDP1 substrate and reaction products were prone to concentration-dependent nucleolytic degradation (Figure 5b, top). Heat exposure, however, led to significant protection, increasing TDP1 reaction products (Figure 5b, bottom).

Unlike TDP2, TDP1 reaction produces 3′-P termini that require phosphatase activity before ligation. To confirm the protective effect of hyperthermia on TDP1 reaction products, we supplemented the reactions with alkaline phosphatase, which converts 3′P to 3′OH termini, resulting in a slower migrating band on denaturing gels. Heat exposure led to a time-dependent increase in 3′-OH termini, which further reinforces the protective effect of hyperthermia from nucleolytic degradation (Figure 5c). Next, we measured full ligation by incubating lysates with the TDP1 substrate in the presence of a complementary duplex substrate, PNK, and ligase. Here again, the formation of complete reaction products, indicative of error-free complete repair, was increased by heat exposure in a time-dependent manner, and the results were significant after only 1 h of heat exposure (Figure 5d–f). If what we observed in vitro is also true in cells, then removing metal ions from cell culture media should also suppress TOP1 mediated DNA strand breaks in both the wild-type and TDP1 knockout cells reminiscent to heat exposure in Figure 3a,b. Indeed, adding EDTA or sodium citrate to the culture media reduced CPT-induced DNA strand breaks (Figure 5g). We noted that the effect of metal chelators was not as strong as heating, which could be dependent on the concentration, time, or the specificity of the chelators. Taken together, we conclude that hyperthermia inhibits nuclease activity, which has two consequences; (1) preventing the nonspecific processing of TOP-induced DNA damage, which reduces the overall quantity of DNA damage (Figure 3), and (2) channeling the repair of the remaining TOP-induced DNA damage to the TDP pathway (Figure 4 and Figure 5).

### 2.4. Hyperthermia Reduces Topoisomerase-Induced Genomic Instability and Inflammation

**Figure 6:**
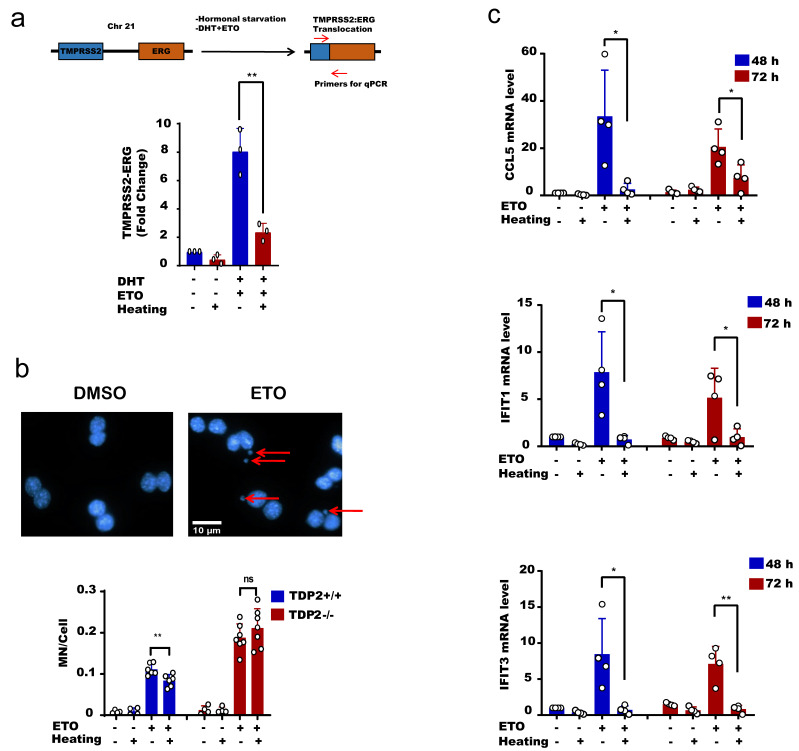
Hyperthermia reduces the level of topoisomerase-induced DNA damage, genomic instability and inflammation. (**a**) LNCaP cells were hormonally starved for 48 h before treatment with hyperthermia at 43 °C for 1.5 h, and then with 100 nM DHT and 100 µM Etoposide (ETO) for 24 h. After treatment, the cells were reincubated for 24 h in fresh media before being harvested. The gene fusion of TMPRSS2:ERG was examined by qPCR. (**b**) MEF^TDP2+/+^ and MFE^TDP2-/-^ cells were treated with DMSO, preheated at 43 °C and/or 15 µM ETO for 1 h, and then cells were synchronized by cytochalasin B for 24 h. After fixation and DAPI staining, the micronuclei (indicated by arrows) were counted in binucleated cells only. (**c**) MEF^TDP2+/+^ cells were treated with DMSO, preheated at 43 °C and/or 30 µM ETO for 2.5 h. The RNA was isolated after 48 or 72 h and the level of CCL5, IFIT1, and IFIT2 were assessed by qPCR. Data are the average of at least three biological replicates ± SD. Asterisks denote statistical significance (* *p* < 0.05, and ** *p* < 0.01; Student’s *t*-test).

The physiological implication of these findings is that hyperthermia favors error-free repair and consequently suppresses hallmarks of genomic instability driven by error-prone nucleases [37,38,39,40,41,42,43]. To test this, we took advantage of the reported TOP2-induced chromosomal translocation models. We used an established protocol to induce chromosomal translocation in the prostate cancer cell line, LNCaP [62]. Hormonally deprived LNCaP cells were treated with etoposide and dihydrotestosterone (DHT) to induce chromosomal translocations, measured by qPCR. Strikingly, heat exposure suppressed TOP2-induced translocations (Figure 6a). Another hallmark of genomic instability is the formation of micronuclei (MN). Here again, heat exposure suppressed the level of etoposide-induced micronuclei from 11.1 MN/100 cells at 37 °C to 8.3 MN/100 cells at 43 °C, ~25% reduction (Figure 6b, left). Notably, the protective effect of hyperthermia on micronuclei was TDP2-dependent, since hyperthermia failed to exert a protective effect in cells lacking TDP2 (Figure 6b, right). Micronuclei are not only a marker of genomic instability but also responsible for DNA damage-induced inflammation. Cyclic GMP-AMP synthase (cGAS) localizes to micronuclei and generates the cyclic dinucleotide cyclic GMP-AMP (cGAMP), which induces a type I interferon response via the adaptor STING, a stimulator of interferon genes [63,64]. Consistent with this, hyperthermia suppressed the etoposide-induced expression of interferon-stimulated genes *IFIT1*, *IFIT3*, and *CCL5*, which lasted for 72 h post-treatment (Figure 6c). Taken together, we conclude that high temperature protects from TOP2-induced genomic instability and inflammation.

## 3. Discussion

Similar to other repair pathways examined to date, we show that the activity of TDP1 and TDP2 is compromised following heat stress. We further show that hyperthermia exhibits a more pronounced effect in inhibiting cellular nucleases, thereby sparing the error-prone removal of TOPcc by nucleases for an error-free repair by TDPs. This leads to suppression of TOP-induced genomic instability, which is consistent with the inverse correlation between cancer incidence and annual temperature in humans [2,3] and mice [65].

Despite their importance, how cells control nuclease activities to prevent detrimental genomic degradation remains unclear [66,67]. Growing evidence suggests that bypassing nuclease activities holds a higher degree of complexity than initially thought. It involves a tight regulation between signaling and repair pathways [68,69,70,71]. Here, we propose a different layer of regulation driven by heat exposure as a physical regulator of the cellular nuclease activity, which results in the suppression of topoisomerase-induced DNA damage and genomic instability.

**Figure 7:**
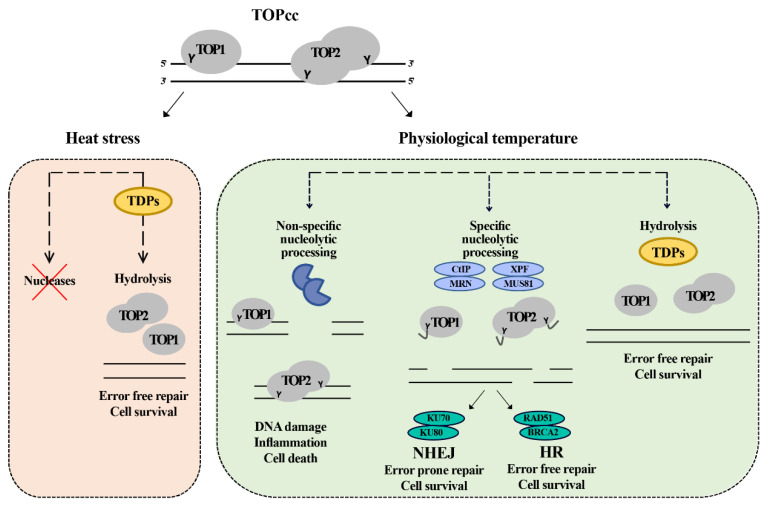
Model for the repair of topoisomerase induced damage in physiological temperature and heat stress. The cell at the normal physiological temperature (37 °C) can employ redundant pathways to remove the trapped topoisomerase. The first pathway is mediated by TDPs enzymes which hydrolyze the tyrosine bond between the topoisomerase and DNA, leading to the removal of the DNA–protein crosslink in error free mode. The second pathway is mediated by specific nucleases (CtIP, MRN, XPF, and MUS81) that nucleolytically cut the DNA, releasing the topoisomerase and a fragment of DNA. This pathway leads to the formation of breaks that can be further repaired by either homologous recombination (HR) or non-homologous end joining (NHEJ) in error free or error prone mode, respectively. The third pathway is cytotoxic and does not lead to the repair of the damage. The cell has an army of nonspecific nucleases. Their toxic involvement can further process the damaged sites leading to the formation of more damage and inducing cell death. Under heat stress, the cell inhibits the nucleases, so it can only depend on TDPs to repair the topoisomerase-induced damage leading to delayed error free repair.

First, we examined the effect of hyperthermia on TDP1 and TDP2 catalytic activities to explain the antagonistic effect between hyperthermia and topoisomerase inhibitors. We showed that TDPs activity was compromised during heat stress. Paradoxically, and only in the presence of metal ions, hyperthermia increased TDP repair products (3′P in the case of TDP1 and 5′P in the case of TDP2). Through a series of biochemical experiments, we showed that this effect was due to hyperthermia-induced inhibition of nucleases. Nuclease inhibition by hyperthermia renders the remaining activity of TDP capable of taking over DNA repair events. Thus, hyperthermia spares the error-prone removal of TOPcc by nucleases for a delayed error-free repair by TDPs (Figure 7).

Several classes of nucleases are metal ion-dependent, and the Mg^2+^ ion is most frequently associated with nucleases due to its abundance, solubility and redox stability [67]. Measuring the topoisomerase-induced DNA damage in the presence of chelators such as EDTA or sodium citrate further confirmed this hypothesis. Prior incubation with chelators reduced the CPT-induced DNA strand breaks similar to hyperthermia. Nonetheless, the current topoisomerase-induced toxicity model relies on the generation of cytotoxic DSBs. DSBs generation can be either direct due to TOPcc collision with replication forks or indirect as a consequence of accumulating positive supercoiling ahead of replication forks due to the inhibition of topoisomerase activity [37,38,49,72]. Our findings suggest that topoisomerase inhibitors induce cytotoxicity through nonspecific processing of TOP-DNA breaks by nucleases. Moreover, several reports suggested that the error-prone repair of topoisomerase-induced DNA breaks lead to the development of many forms of cancer [37,41,42,43,49,51,52,53,54]. Our findings suggest that hyperthermia protects from TOP-induced genomic instability by reducing the quantity of DNA damage and by favoring an error-free repair. Hyperthermia effectively shielded the DNA from etoposide-induced chromosomal translocations in a prostate cancer model and suppressed the formation of etoposide-induced micronuclei and inflammation.

Why nucleases are more labile to hyperthermia than other proteins such as TDPs is unclear. How the repair of TOPcc is regulated in disorders with impaired thermoregulatory autonomic pathways will be exciting to explore [7]. Furthermore, our model has implications for viral infections. The infection-induced fever may increase viral load in the host by inhibiting the nucleases, giving the virus a chance to replicate its genome using TDP2, which has been shown to facilitate viral replication [73,74].

In summary, we show that high temperature impacts topoisomerase-induced DNA damage by: (1) reducing the extent of DNA strand breaks by inhibiting the nucleases and (2) enhancing the quality of repair of the remaining damage by a more pronounced inhibitory effect on nucleases, favoring the TDP error-free pathways. These findings may help to explain the inverse relationship between environmental temperature and the geographical distribution of cancer and could be exploited in the applications of controlled heat as an adjunct to topoisomerase targeting therapeutics.

## 4. Methodology

### 4.1. Cell Culture, Synchronization, and Hyperthermia Treatment

Colorectal cancer cell line, RKO, lymphoblast cell lines, TK6^TDP1+/+^ and TK6^TDP1-/-^, and prostate cancer cell line, LNCaP, were cultured in standard RPMI-1640 media (Lonza, USA). Transformed mouse embryonic fibroblasts MEF^TDP2+/+^, MFE^TDP2-/-^, and breast cancer cell line, MCF-7 were cultured in DMEM media (Lonza, USA). Both media were supplemented with 10% FBS (Sigma, USA) and 1% penicillin/streptomycin (Lonza, USA). The cells were maintained at 37 °C in 5% CO_2_ incubator. For cellular synchronization double-thymidine block was used by treatment with 2 mM thymidine for 16 h, then released for 6 h in fresh media, and finally treated with thymidine for another 16 h. Hyperthermia treatment was applied by maintaining cells at 37, 43, or 45 °C in CO_2_ incubators (for long term treatment) or water bath (short term treatment). All cell lines were obtained from the University of Sheffield (El-Khamisy lab) and routinely tested negative for mycoplasma.

### 4.2. Comet Assays

Alkaline comet assay measures SSBs and DSBs, whereas neutral comet assays measure DSBs. After different treatments, cells were collected, washed by cold PBS and suspended in PBS. Equal volume of 1.3% low melting agarose (Type VII, Sigma, USA) was added to the cells. The mixture was layered onto prechilled frosted glass slides (Fisher Scientific, USA), pre-coated with 0.6% agarose. For alkaline comet assays, lysis buffer (2.5 M NaCl, 10 mM Tris-HCl pH 7, 100 mM EDTA, 1% Triton X-100, 10% DMSO, pH 10) was added on the slides for 1 h at 4 °C. Electrophoresis was performed in 50 mM NaOH and 1 mM EDTA at 25 V for 30 min. For neutral assays, lysis buffer (2.5 M NaCl, 10 mM Tris-HCl pH 7, 0.1 M EDTA, 0.5% Triton X-100, 3% DMSO, 1% N-lauroylsarcosine, pH 9.5) was added for 90 min at 4 °C, and electrophoresis was performed in 0.3 M sodium acetate, and 100 mM Tris-HCl for 1 h. The samples were neutralized in 400 mM Tris-HCl pH 7, and DNA was stained with SYBR Green I (1:10,000, in PBS) for 10 min. Average tail moments from at least 100 cells for each sample were detected using Comet Assay IV software (Perceptive Instruments, UK). Data were presented as an average ± SD from three independent experiments. Statistical analysis was performed using Student’s *t*-test.

### 4.3. Cell Lysate Preparation

After treatment, the cells were collected and lysed in lysis buffer (40 mM tris-HCL PH 7.5, 1% Triton X-100, 100 mM NaCl, 1 mM DTT, 10% sigma protease inhibitor cocktail). Total protein concentration was determined using the Bradford assay. The whole-cell lysate was kept at −80 °C.

### 4.4. TDPs Activity Assay

The in vitro 3′-tyrosyl-DNA phosphodiesterase and 5′-tyrosyl-DNA phosphodiesterase activities were determined using a gel-based assay. Biochemical assays were performed in 10 μL reaction volumes containing TDP1 reaction buffer (50 mM Tris HCL pH 7.5, 50 mM KCl, 1 mM DTT, 100 μg/mL BSA, and 10 M EDTA or 1 mM MgCL2) or TDP2 reaction buffer (50 mM Tris HCL pH 7.5, 50 mM KCl, 1 mM DTT, 100 μg/mL BSA, and 1 mM MgCl2), cell lysate, and 6 nM Cy5.5-labelled substrate oligomer. TDP1 substrate 5′Cy5.5-GATCTAAAAGACT (3′P-Tyr) (Midland Certified Reagent Company, USA) was used to determine TDP1 catalytic activity. Only in the ligation experiments, the TDP1 substrate was annealed to 5′-GATGAGTCTTTTAGATC-3′, and both are ligated to the annealed complementary (5′-ATGGTAGGCAAC-3′ and 5′-CATCGTTGCCTACCAT-3′). For TDP2 reaction, 5′(P-Tyr) CATCGTTGCCTACCAT (Cy5.5)-3′ (Midland Certified Reagent Company, USA) complemented to 5′-ATGGTAGGCAAC-3′ to form 4 nucleotide overhang was used. In case of the ligation reaction, this complement was ligated to the annealed complement of (5′-GATCTAAAAGACT-3′, and 5′-GATGAGTCTTTTAGATC-3′). The reactions progressed at 37 °C for 30–90 min and were quenched with 10 μL loading buffer (44% deionized formamide, 2.25 mM Tris-borate, 0.05 mM EDTA, and 1% bromophenol blue). Samples were then heated at 90 °C for 5 min before separation on a 20% urea SequaGel (Geneflow, UK) at 150 V. Reaction products were visualized by gel imaging using ChemiDoc™ MP Imaging System (Bio-Rad, USA).

### 4.5. Western Blot

After treatment, cells were washed, collected and lysed in RIPA buffer. After that, equal amounts of total protein were boiled with SDS-polyacrylamide gel electrophoresis (PAGE) sample buffer at 95 °C for 5 min and 40–90 µg of protein was loaded for separation by 12% SDS-PAGE. Electrophoresed proteins were then transferred onto PVDF membranes (Millipore, USA) either using semidry (turbo, Bio-Rad, USA) or wet transfer. The membranes faced blocking in 5% non-fat milk for 1 h. Then, the membranes were incubated with primary antibodies; anti-TDP1 (Santa Cruz, USA, SC-365674), anti-TDP2 (Santa Cruz, USA, SC-377280), anti-UCHL3 (Thermo Fisher, MA517230) overnight at 4 °C or anti-GAPDH (Abcam, ab37168) for 1 h at room temperature. After three washes in T-PBS (3 × 5 min), membranes were then incubated with anti-mouse horseradish peroxidase-linked IgG antibody (Thermo Fisher Scientific, USA) for 1 h at room temperature. After 3 × 5 min washes in T-PBS, visualization was performed by enhanced chemiluminescence (ECL) detection reagent (Amersham ECL Reagent, GE, USA). Imaging was performed by ChemiDoc™ MP (Bio-Rad, USA). The raw uncropped images wre included in supplementary Figure S4.

### 4.6. Viability

TK6^TDP1+/+^, and TK6^TDP1-/-^ cells (5000 cells/well) were treated with CPT at varying concentrations in triplicate after hyperthermia treatment. On day three, 20 μL cell-titer blue (Promega, USA) was added, and the plate was incubated at 37 °C. After 4 h, the viability was quantified as fluorescence intensity using a microplate reader, FLUOstar Omega (BMG LABTECH). In the case of MEF^TDP2+/+^ and MFE^TDP2-/-^ cells (5000 cells/well) were treated with varying etoposide concentrations after hyperthermia treatment. On day 5, cell-titer blue (Promega, USA) was added, and the viability was measured.

### 4.7. TOP1 Activity Assay

DNA topoisomerase I activity was assayed by the relaxation of negatively supercoiled plasmid DNA as described previously [75]. The reaction was done in a total volume of 20 µL containing 50 mM Tris-HCl, pH 7.5, 100 mM KCl, 1 mM DTT, 10 mM EDTA, 5 µg/mL BSA, and 200 ng of supercoiled pEGFPN1 plasmid. After incubation at 37 °C for 30 min, the reactions were stopped by adding 5 µL of loading dye (50 mM EDTA, 50% glycerol, and 0.5% bromophenol blue). The DNA was electrophoresed in a 1% agarose gel with TAE buffer (50 mM Tris–HCl, pH 8.0, and 2 mM EDTA). The gels were visualized under UV illumination after being stained with ethidium bromide.

### 4.8. Quantitative PCR

Cells were washed and collected for RNA extraction after each treatment according to the manufacturer’s instructions (RNeasy Mini Kit; Qiagen, Germany). High-Capacity cDNA Reverse Transcription Kit (Thermo Fisher Scientific, USA) was used to prepare the cDNA. The qPCR (QuantStudio 12K Flex Real-Time PCR System, Thermo Scientific, USA) was performed using SYBR Green Master Mix (Thermo Fisher Scientific, USA) and the following primers: TDP1 F: 5′-CAGAGTTCAGGAAGAAGCCAATC-3′; TDP1 R: 5′-GCATCATTTTCGTGTGGTGTGTTC-3′; TDP2 F: 5′-AGCCCAAGACCTATGTTGACC-3′; TDP2 R: 5′-CTAAGTAGGAACACACCCCTC-3′; GAPDH F: 5′-TTCGTCATGGGTGTGAACCA-3′; GAPDH R: 5′-TGATGGCATGGACTGTGGTC-3′; mouse IFIT1 F: 5′-TCTAAACAGGGCCTTGCAG-3’; mouse IFIT1 R: 5′-GCAGAGCCCTTTTTGATAATGT-3’; mouse IFIT3 F: 5′-TGAACTGCTCAGCCCACA-3′; mouse IFIT3 R: 5′-TCCCGGTTGACCTCACTC-3’; mouse CCL5 F: 5′-ACGTCAAGGAGTATTTCTACAC-3′; mouse CCL5 R: 5′-GATGTATTCTTGAACCCACT-3′; mouse GAPDH F: 5′-AACTTTGGCATTGTGGAAGG-3′; mouse GAPDH R: 5′-ACACATTGGGGGTAGGAACA-3′.

### 4.9. Chromosomal Translocation Assay

We adapted a published technique for the induction of chromosomal translocation in a prostate cancer cell line [62]. Briefly, LNCaP cells were starved in phenol-free DMEM (Lonza, USA) supplemented with 5% charcoal-stripped fetal bovine serum (Biowest, France) before treatment with hyperthermia at 43 °C for 1.5 h, and then with 100 nM dihydrotestosterone (DHT) and 100µM etoposide for 24 h. After treatment, the cells were reincubated for 24 h in fresh media before being harvested. Cells were then collected for RNA extraction according to the manufacturer’s instructions (RNeasy Mini Kit; Qiagen, Qiagen, Germany). High-Capacity cDNA Reverse Transcription Kit (Thermo Fisher Scientific, USA) was used to prepare the cDNA. The gene fusion events of TMPRSS2:ERG were examined by qPCR (QuantStudio 12K Flex Real-Time PCR System, Thermo Scientific) using SYBR Green Master Mix (Thermo Fisher Scientific, USA). Relative quantities of TMPRSS2:ERG fusion transcript was normalized to GAPDH and the expression level of TMPRSS2. The relative amount of each fusion transcript was then calibrated to the DHT sample. The used primers are TMPRSS2 F: 5′-CTGGTGGCTGATAGGGGAT-3′; TMPRSS2 R: 5′-GTCTGCCCTCATTTGTCGAT-3′; TMPRSS2-ERG F: 5′-AGCGCGGCAGGTTATTCCA-3′; TMPRSS2-ERG R: 5′-ATCATGTCCTTCAGTAAGCCA-3′.

### 4.10. Micronuclei

Micronuclei were analyzed in transformed MEFs previously seeded onto coverslips. Following treatment, cytochalasin B was added at 4 mg/mL. After 24 h, cells were fixed, stained with DAPI, and images were acquired by IN Cell Analyzer 2200 (GE Healthcare Life Sciences, USA). Only binucleated cells were scored.

### 4.11. Immunoprecipitation and Nano-LC MS/MS Analysis

TK6 cells were mock-treated or treated with 10 µM MG132 and/or heated at 43 °C for 1.5 h. Cells were washed with cold PBS, lysed on ice in RIPA lysis buffer (50 mM Tris, pH 7, 150 mM NaCl, 0.5% sodium deoxycholate and 1% NP-40) supplemented with a protease inhibitor cocktail (Sigma, USA) for 1 h, and then centrifuged at 15,000 rpm for 10 min. The supernatant was mixed with protein A/G-Sepharose mix (Santa Cruze, USA), preswelled in PBS and precoated with anti-human TDP1 antibodies by gently shaking for 1 h at 40 °C and centrifuged for 1 min at 3000 rpm. After washing with lysis buffer, the immunocomplex was washed three times with lysis buffer. TDP1 immunoprecipitate were subjected to protein digestion and stage tipping as described [76]. Nano-LC MS/MS analysis was carried out using TripleTOF 5600 + (AB Sciex, Canada) interfaced at the front end with Eksigent nanoLC 400 auto-sampler with Ekspert nanoLC 425 pump. CHROMXP C18CL 5 μm (10 × 0.5 mm) (Sciex, Germany). Column was used to trap the peptides in trap and elute mode. The MS and MS/MS ranges were 400–1250 and 170–1500 *m*/*z*, respectively. A 55 min linear gradient of 3–40% solution B (80% ACN, 0.2% formic acid) was applied. Data-dependent acquisition (DDA) mode with a charge state of 2–5 was used to select the 40 most intense ions sequentially [76]. MS/MS spectra were searched using X Tandem in Peptide shaker (version 1.16.26) against Homo Spaiens (Taxon identifier: 9606) UniProtKB/TrEMBL database (213,287 entries) with reversed decoy sequences and FDR of 1%. Biomart was used to map the UniProtKB Gene Name ID to gene names and gene description [77,78]. IDs that were not identified via Biomart were manually added.

## 5. Conclusions

Hyperthermia enhances the anticancer effects of radiotherapy and multiple chemotherapeutics. In a remarkable contrast, hyperthermia protects cells from topoisomerase-targeting therapeutics through an unknown mechanism. Here, we report that hyperthermia inhibits the error-prone nucleases and the error-free TDPs pathways. The inhibitory effect of hyperthermia is more pronounced on nucleases, thereby favoring repair by the error-free TDP pathways, which reduces hallmarks of therapy-associated chromosomal instability.

## Data Availability

All data are contained within the article and supplementary material.

## Supplementary Materials

The following are available online at https://www.mdpi.com/article/10.3390/cancers13102315/s1, Figure S1: The effect of hyperthermia on TDP1 and UCHL3. Figure S2: Hyperthermia reduces topoisomerase-induced-DNA damage independent of topoisomerase catalytic activity and heat shock proteins. Figure S3: The effect of hyperthermia on TDP1 and TDP2 activity. Figure S4: Uncropped Western blot images. Table S1: Mass spec raw data.
