# Supplementary material for: High Temperature Drives Topoisomerase Mediated Chromosomal Break Repair Pathway Choice
Source: Cancers (Basel). 2021 May 12;13(10):2315. doi: 10.3390/cancers13102315 (PMC8151962; doi:10.3390/cancers13102315)
Supplement: Supplementary file 1 [file cancers-13-02315-s001.zip › cancers-1171904-supplementary-FINAL/Figure S1 V20.pdf]

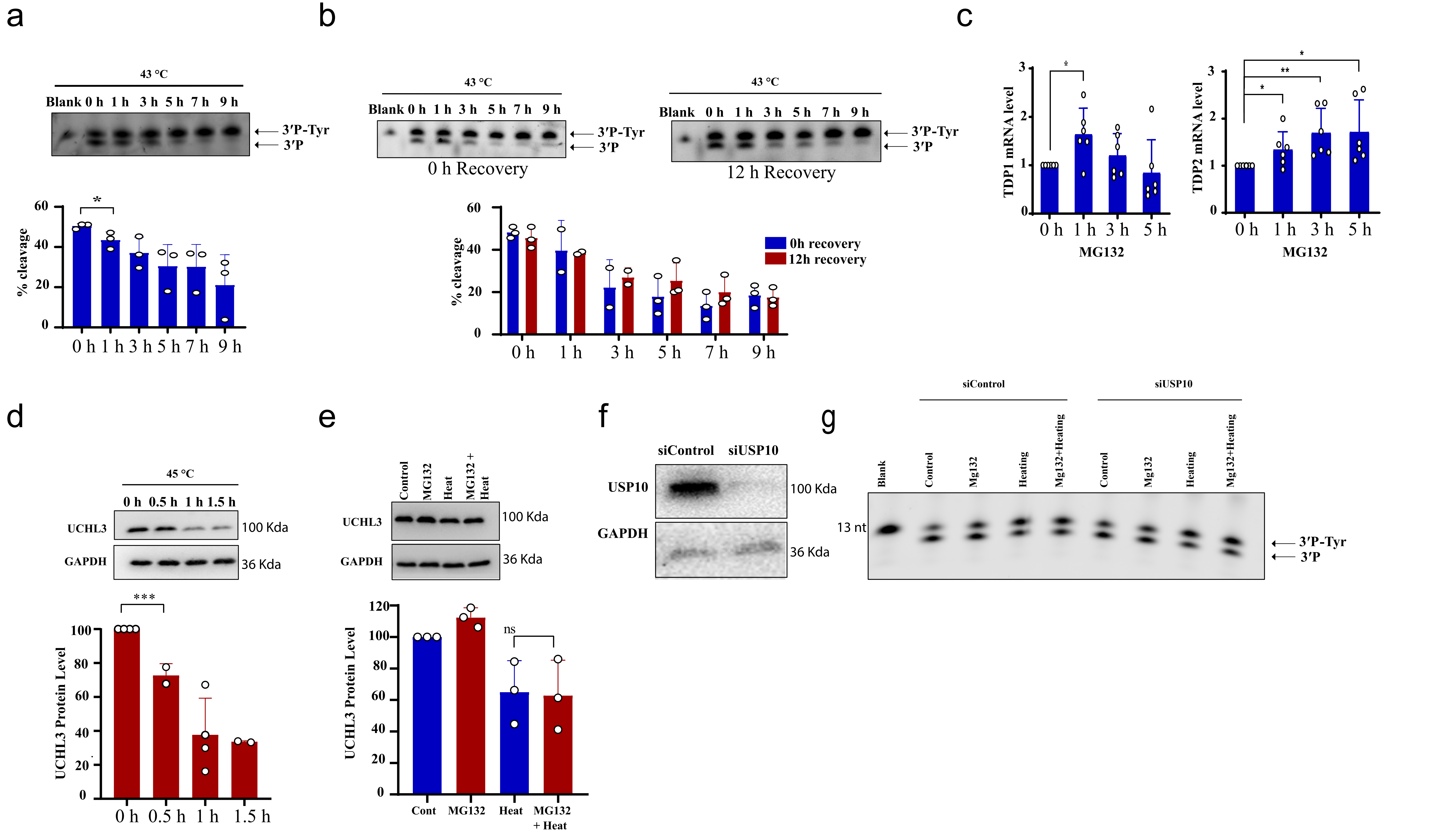

Supplementary Fig 2: The effect of hyperthermia on TDP1 and UCHL3. a) MCF-7 cells were incubated for the indicated time at 43°C and whole cell lysates (0.2 μg) were incubated with TDP1 substrate for 0.5h to monitor TDP1 catalytic activity in buffer containing EDTA. Reaction products were separated by 20% denaturing PAGE. TDP1 catalytic activity was quantified as % cleavage of 3’-PY to 3’-P. b) RKO cells were incubated for the indicated time at 43°C, and cells collected (directly or after 12h recovery at 37°C), lysed and incubated with TDP1 substrate for 0.5h as in (a). c) RNA was isolated from RKO cells that were treated with 10 μM MG132 for the indicated time, and qPCR was used to determine the level of both the TDP1 and TDP2 mRNA.d) Western blot was used to determine the level of UCHL3 after incubating TK6 cells at 45°C for the indicated time points e) Western blots showing the level of UCHL3 after incubating TK6 cells with DMSO (control), 10 μM MG132 and/or heating at 45°C. f) Validation of USP10 silencing in RKO cells by Western blot. g) RKO cells were untreated, treated with 10 μM MG132 for 3h, treated with heating at 43 0C for 3h, or treated with both MG132 and hyperthermia. Then the cells were lysed and incubated with TDP1 substrate to assess TDP1 activity as in (A). Data are the average of three biological replicates ± STD. Asterisks denote statistical significance (*P<0.05, **P<0.01 and ***P<0.001 by student t-test)
