# Supplementary material for: High Temperature Drives Topoisomerase Mediated Chromosomal Break Repair Pathway Choice
Source: Cancers (Basel). 2021 May 12;13(10):2315. doi: 10.3390/cancers13102315 (PMC8151962; doi:10.3390/cancers13102315)
Supplement: Supplementary file 1 [file cancers-13-02315-s001.zip › cancers-1171904-supplementary-FINAL/Figure S2 V20.pdf]

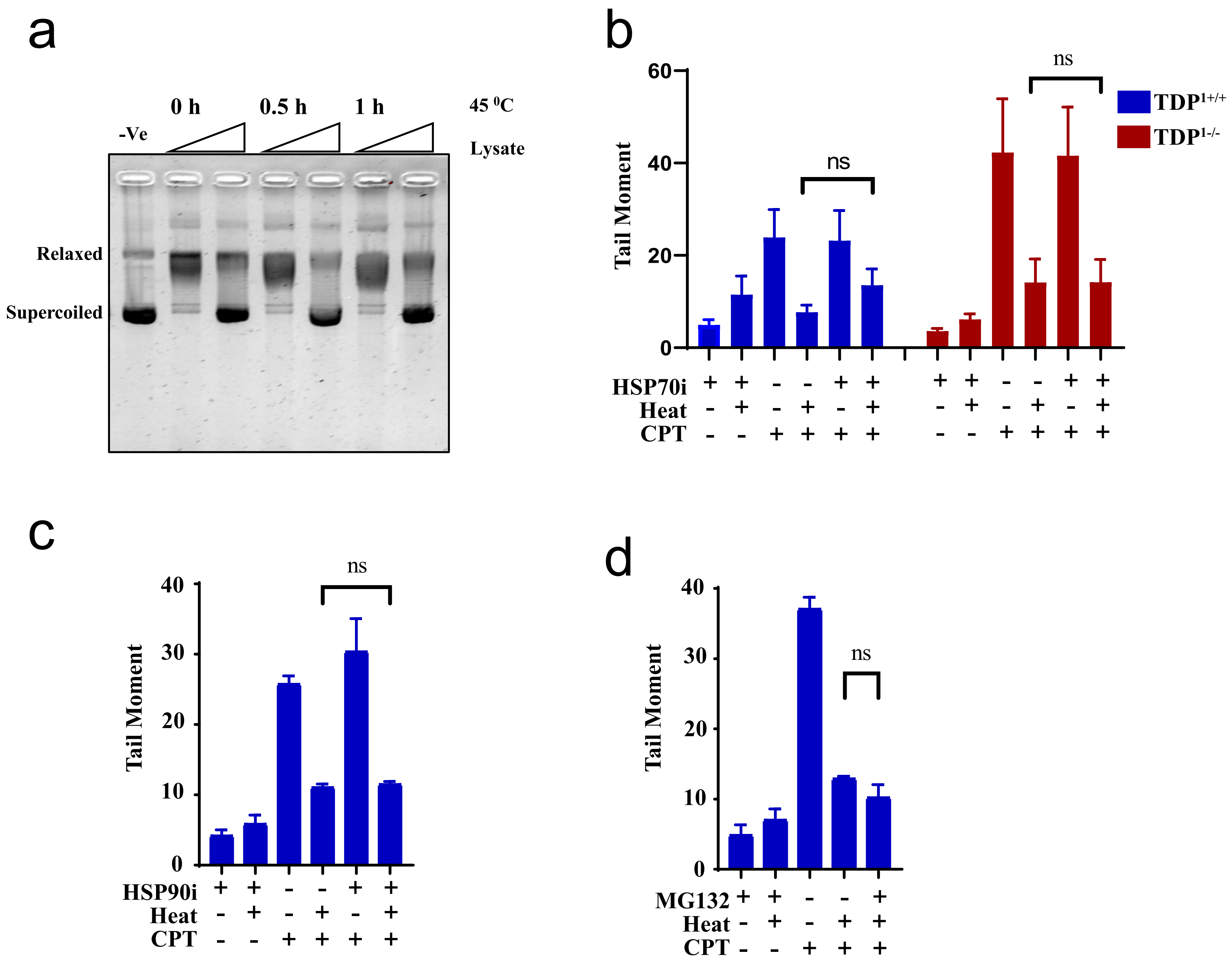

Supplementary Fig 1: Hyperthermia reduces topoisomerase induced DNA damage independent of topoisomerase catalytic activity and heat shock proteins.
a) RKO cells were incubated at 45°C for the indicated time, and then the cells were collected and lysed. Whole cell lysates were used as a source for topoisomerase I activity. Topoisomerase I activity was assayed by the relaxation of negatively supercoiled plasmid, pEGFPN1. b) G1 synchronized TK6 wild-type and TDP1 KO cells were incubated at 37°C or 43°C for 3h, then 100 μM CPT and/or 60 μM HSP70i (pifithrin-u) was added and DNA strand breaks quantified by alkaline comet assays. c) G1 synchronized TK6 cells were incubated at 37°C or 43°C for 3h, followed by100 μM CPT, and/or 25 μM HSP90i (17-AAG) and strand breaks quantified by alkaline comet assays. d) TK6 cells were incubated at 37°C or 43°C for 3h, ollowed by 100 μM CPT and/or 10 μM MG132, and DNA strand breaks quantified by the alkaline comet assay. Data is a representative for three ibdependent repeats.
