# Supplementary material for: High Temperature Drives Topoisomerase Mediated Chromosomal Break Repair Pathway Choice
Source: Cancers (Basel). 2021 May 12;13(10):2315. doi: 10.3390/cancers13102315 (PMC8151962; doi:10.3390/cancers13102315)
Supplement: Supplementary file 1 [file cancers-13-02315-s001.zip › cancers-1171904-supplementary-FINAL/Figure S3 V20.pdf]

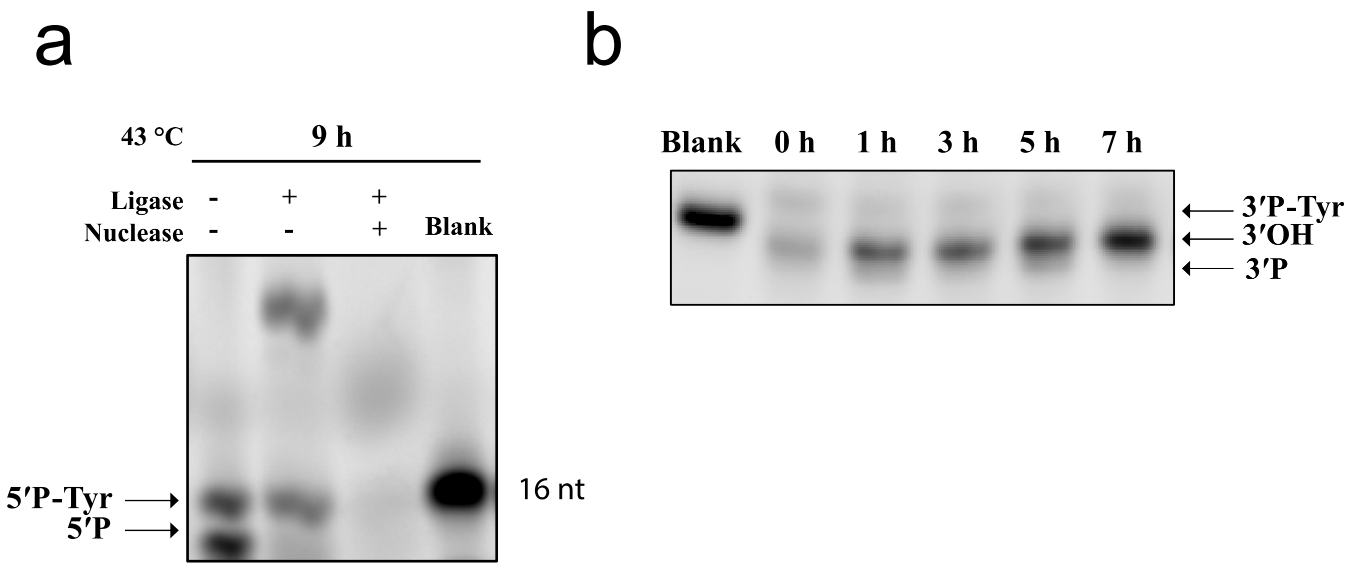

Supplementary Fig 3: The effect of hyperthermia on TDP1 and TDP2 activity. a) Treatment of whole cell lysates from RKO cells with nuclease cocktail reversed the protective effect of 9h heating at 43°C on TDP2-substrate, TDP2-product, and the ligatable product. b) RKO cells were incubated at 43°C for the indicated time and cell lysates incubated with TDP1 substrate for 0.5h to monitor TDP1 catalytic activity in a buffer containing Mg2+. Reaction products were separated by 20% denaturing PAGE.
