# Supplementary material for: High Temperature Drives Topoisomerase Mediated Chromosomal Break Repair Pathway Choice
Source: Cancers (Basel). 2021 May 12;13(10):2315. doi: 10.3390/cancers13102315 (PMC8151962; doi:10.3390/cancers13102315)
Supplement: Supplementary file 1 [file cancers-13-02315-s001.zip › cancers-1171904-supplementary-FINAL/Figure S4 uncropped WB images.pdf]

Fig1A: TDP1 invitro assay

Blank, 0h, 1h, 3h, 5h, 7h, 9h

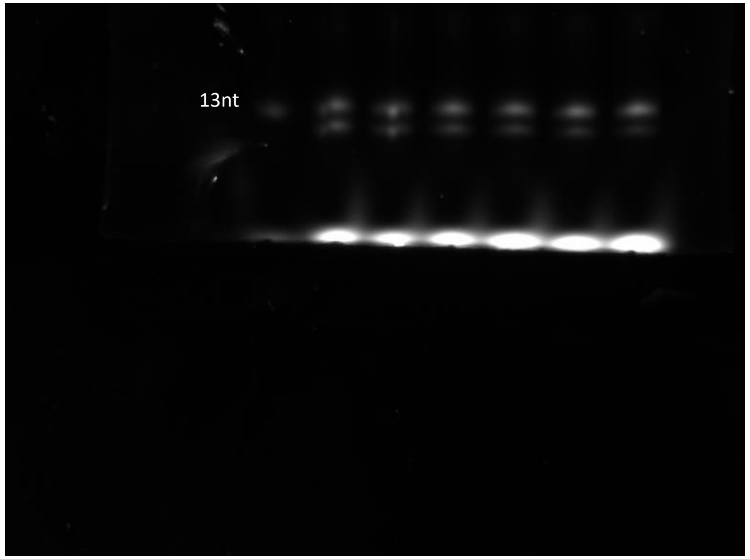

Blank, 0h, 1h, 3h, 5h, 7h, 9h

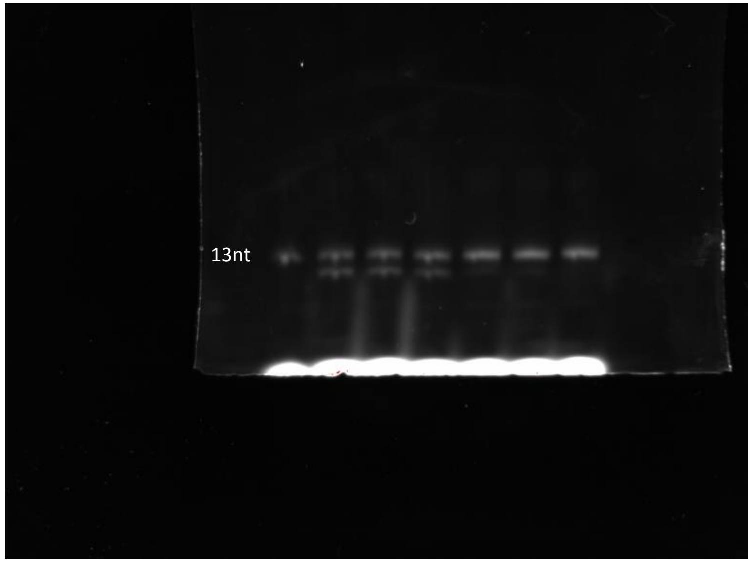

Blank, 0h, 1h, 3h, 5h, 7h, 9h

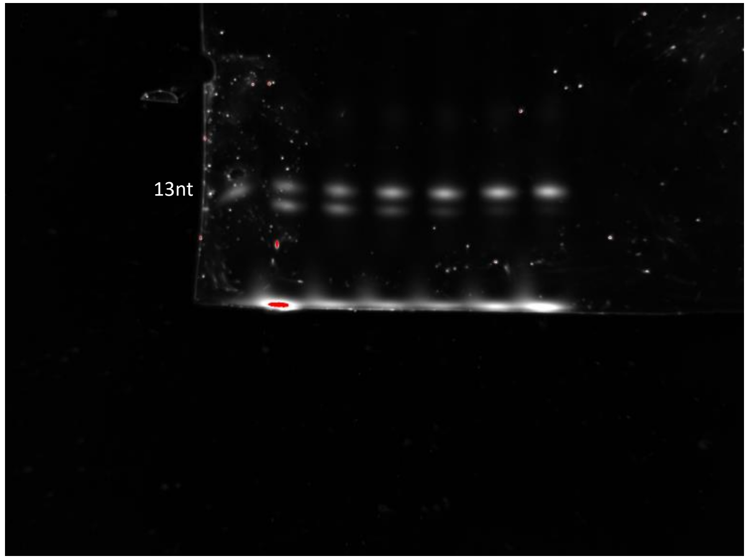

**% cleavage**	**0h**	**1h**	**3h**	**5h**	**7h**	**9h**
**Repeat1**	**44.1074**	**40.25582**	**35.72831**	**20.84586**	**24.4857**	**8.377683**
**Repeat2**	**50.86411**	**41.85025**	**26.33043**	**21.4663**	**18.47522**	**15.13043**
**Repeat3**	**45.46976**	**36.36732**	**31.3317**	**29.97317**	**24.6808**	**19.03491**

Fig1B: TDP1 Western

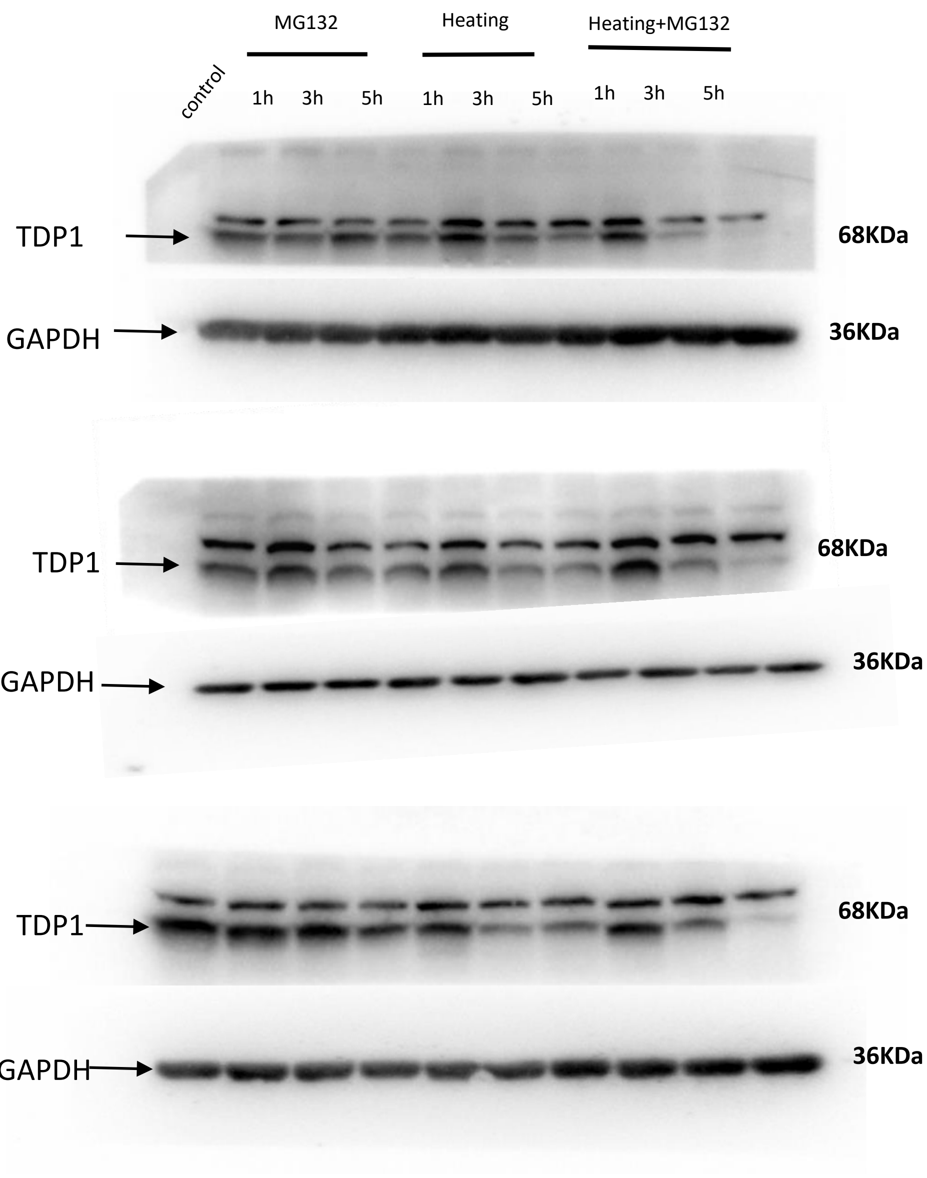

%Normalized volume intensity			
	**Repeat1**	**Repeat2**	**Repeat3**
**control**	100	100	100
**MG132 1h**	74.83055121	100.7597	80.101
**MG132 3h**	85.58599175	80.70128	89.05894
**MG132 5h**	63.57478614	70.82019	71.99743
**heating 1h**	73.83334011	88.92883	81.48503
**heating 3h**	51.42533887	39.20809	53.65322
**heating 5h**	51.82419206	52.79384	51.46706
**MG132 + heating 1h**	89.18765113	95.56353	70.39322
**MG132 + heating 3h**	30.75925353	69.93904	41.86297
**MG132 + heating 5h**	0	38.15382	14.06006

Fig1C: TDP1 invitro assay

MG132

0h 1h 3h 5h 7h 9h blank

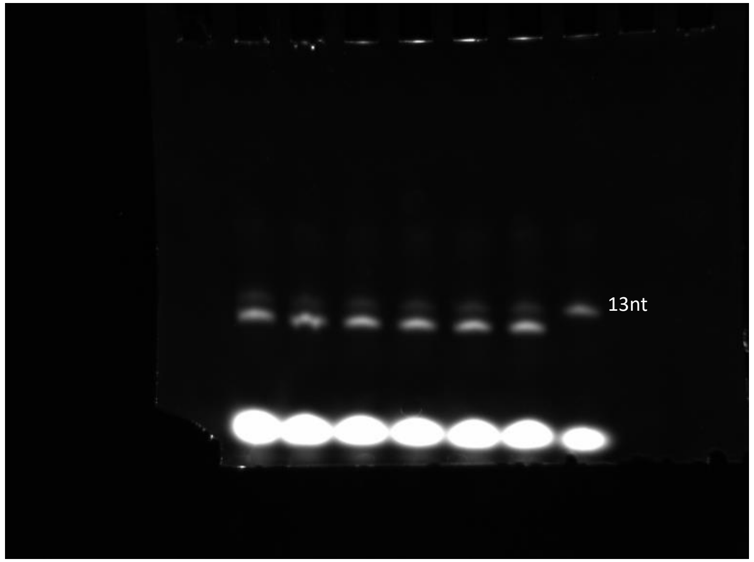

Heating

0h 1h 3h 5h 7h 9h blank

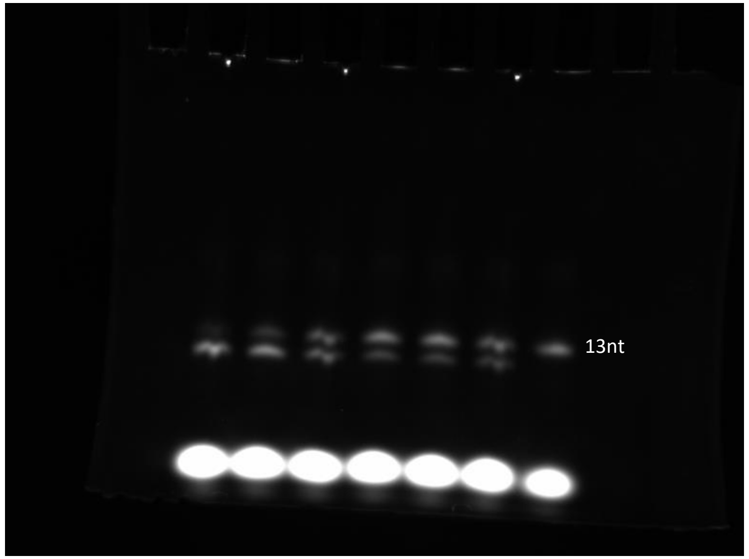

MG132+Heating

0h 1h 3h 5h 7h 9h blank

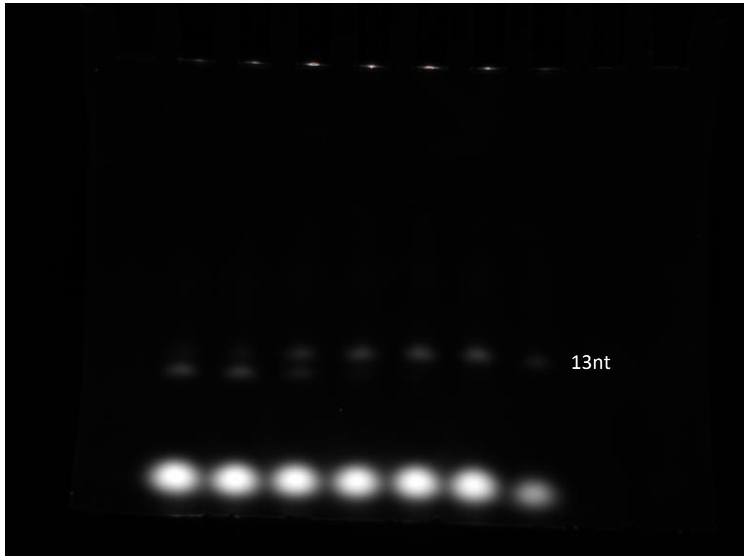

Repeat1

Fig1C: TDP1 invitro assay continue

MG132 | Heating | MG132+Heating

0h 1h 3h 5h 7h 9h blank | 0h 1h 3h 5h 7h 9h blank | 0h 1h 3h 5h 7h 9h blank

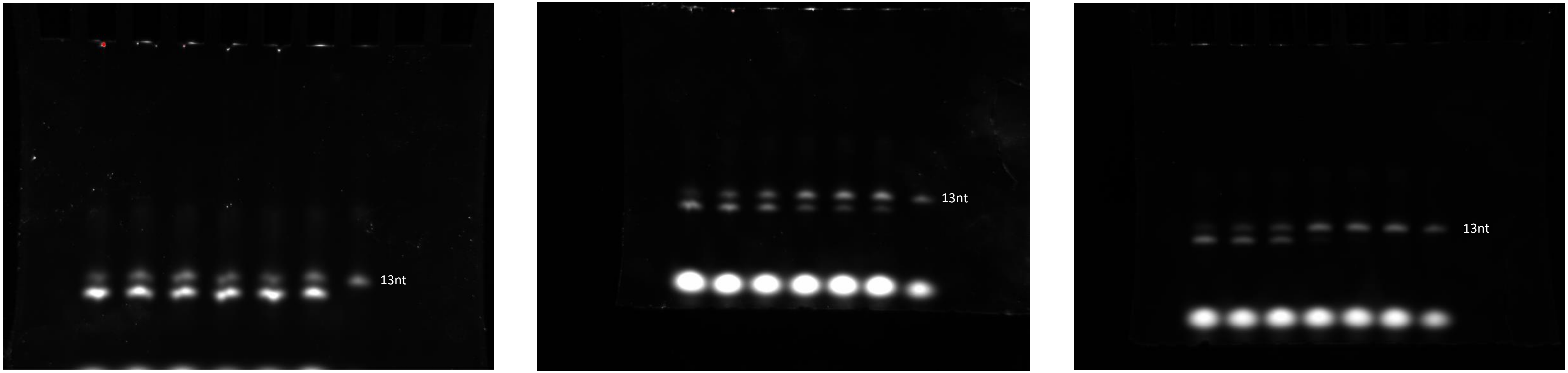

Repeat2

Fig1C: TDP1 invitro assay continue

MG132

0h 1h 3h 5h 7h 9h blank

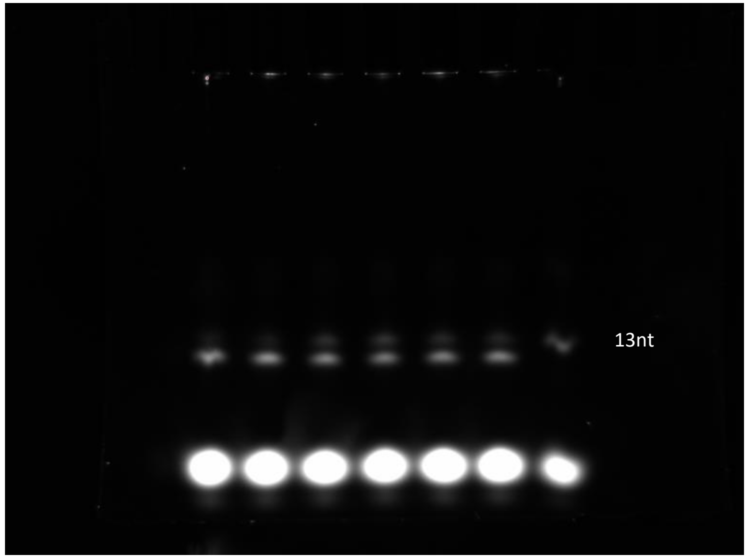

Heating

0h 1h 3h 5h 7h 9h blank

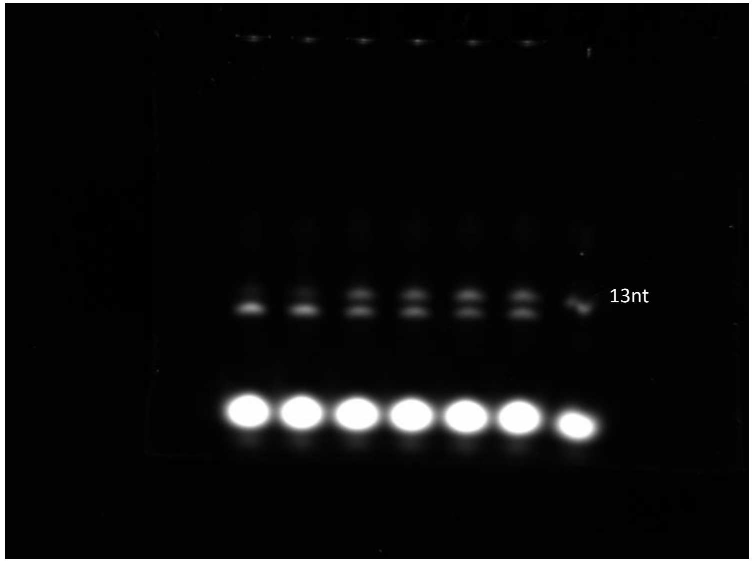

MG132+Heating

0h 1h 3h 5h 7h 9h blank

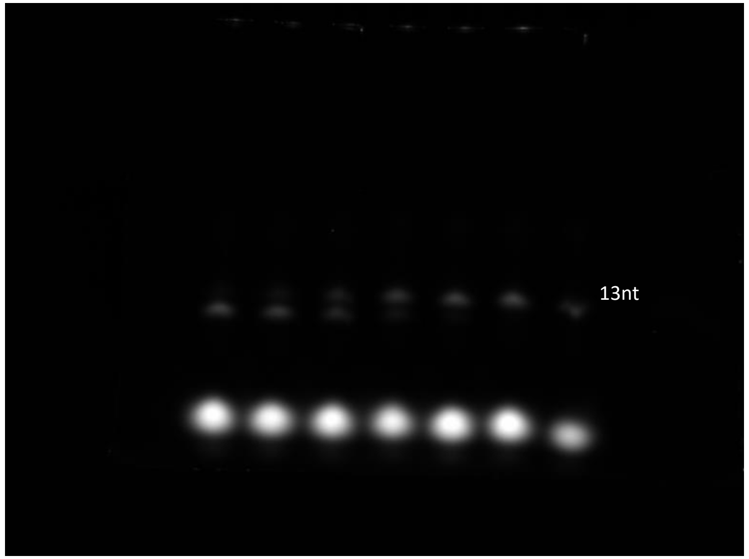

Repeat3

Fig1C: TDP1 invitro assay continue

MG132

0h 1h 3h 5h 7h 9h blank

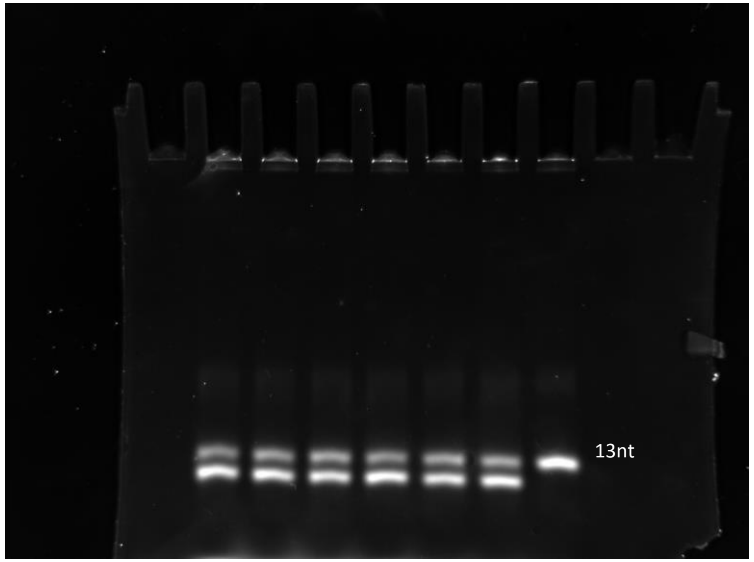

Heating

0h 1h 3h 5h 7h 9h blank

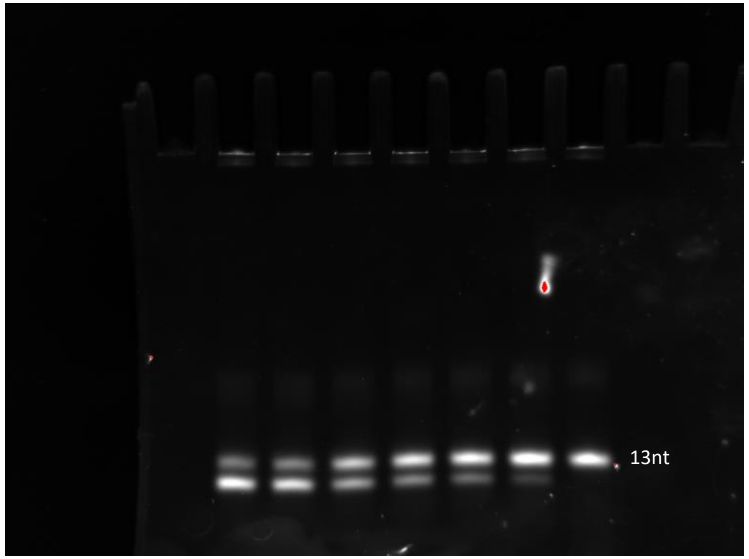

MG132+Heating

0h 1h 3h 5h 7h 9h blank

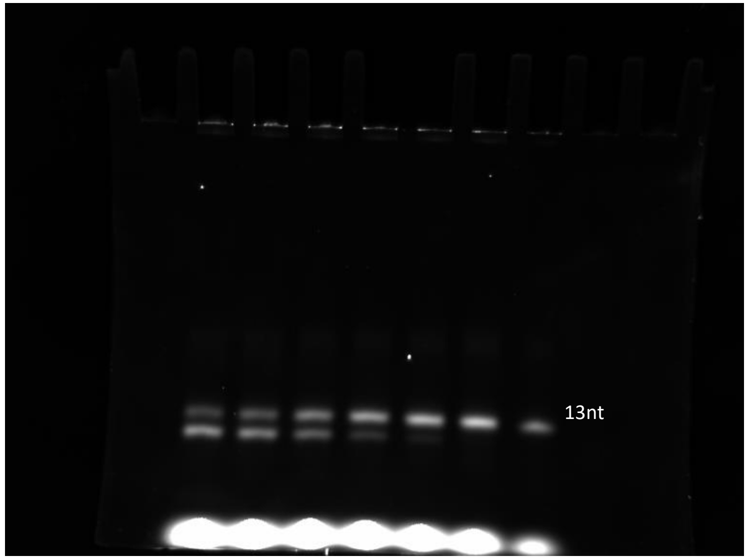

Repeat4

Fig1C: TDP1 invitro assay continue

%Cleavage	MG132				Heating				MG132+Heating			
	repeat1	repeat2	repeat3	repeat4	repeat1	repeat2	repeat3	repeat4	repeat1	repeat2	repeat3	repeat4
0h	100	100	100	100	100	100	100	100	100	100	100	100
1h	98.0127936	90.0221765	98.9850136	99.9657555	84.3564706	77.443618	94.8784748	92.4317097	97.9481551	79.1070881	82.338792	84.7184769
3h	96.4679745	82.6414874	85.6441555	93.3848144	56.4518476	65.8744549	61.0784494	60.4494652	43.0599357	61.8999434	57.792873	55.526105
5h	96.3743783	90.9826493	80.0676572	98.1406558	34.0858122	32.8051754	59.4524321	44.6881222	9.80977673	9.45700086	21.41873	24.7090609
7h	93.894282	95.6714469	88.4287951	92.8622565	31.2403941	26.368722	46.5971514	33.4912353	0	0.74925155	6.84279	6.92824291
9h	94.541549	88.3246333	91.1775883	98.6155145	37.0692539	21.0936803	55.0011702	13.4156969	0	0	0	0

Fig1D: TDP1 invitro assay

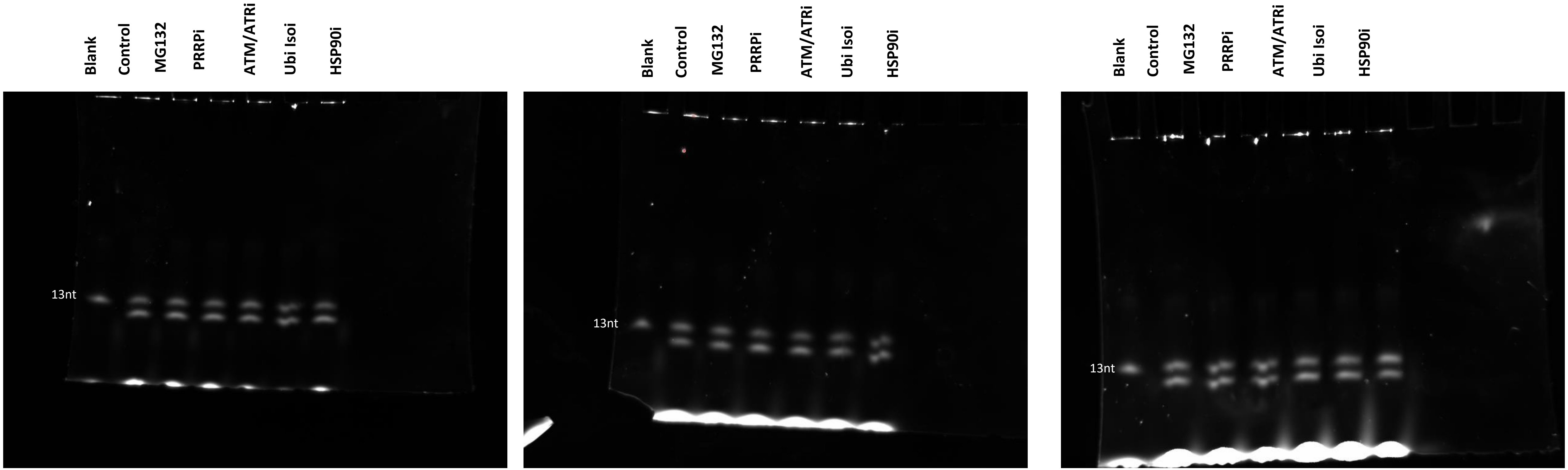

Repet1

Repeat2

Repeat3

Fig1D: TDP1 invitro assay, continue

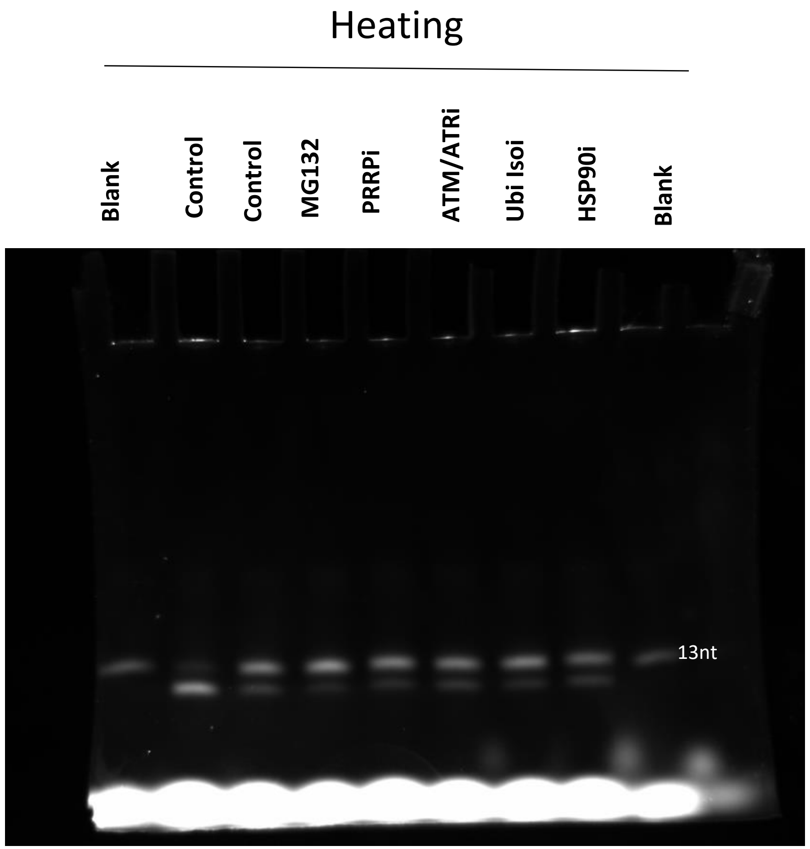

Repet1

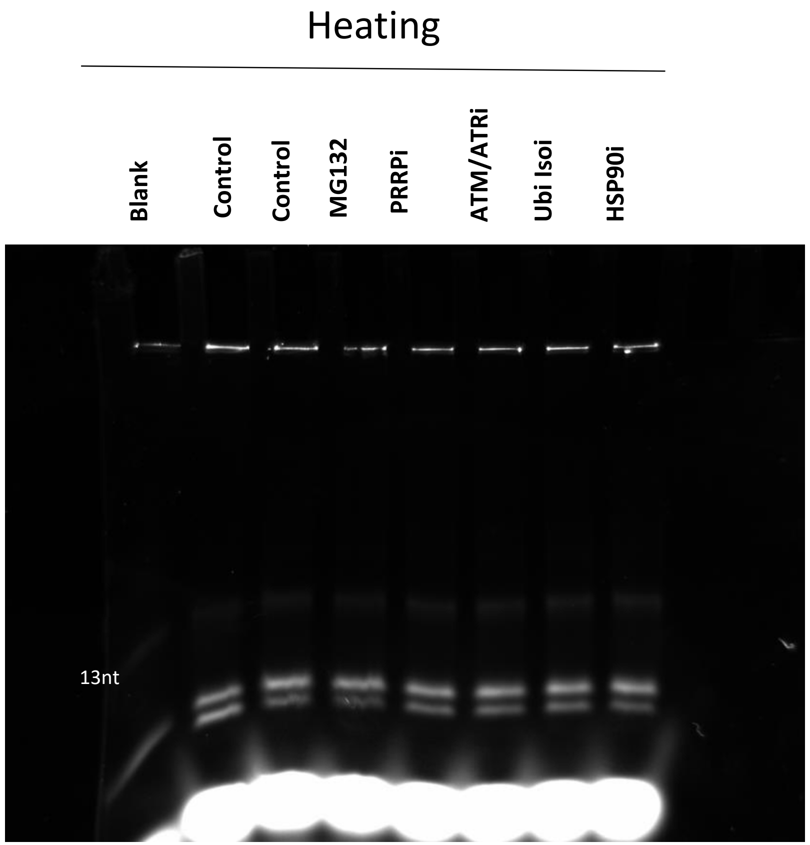

Repeat2

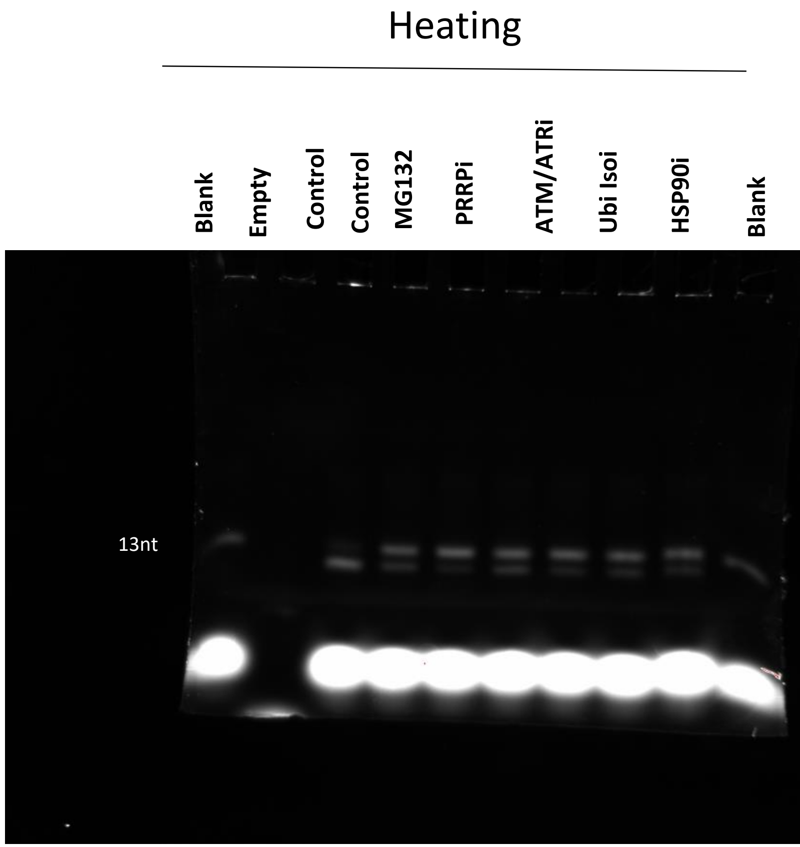

Repeat3

Fig1D: TDP1 invitro assay, continue

%cleaveage	37 C							43 C					
	Control	Proteasomei	PARP1i	ATM/ATRi	Ubi Isoi	HSP90i		Control	Proteasomei	PARP1i	ATM/ATRi	Ubi Isoi	HSP90i
Repeat1	57.20786	56.74524	59.02905	53.23292	57.12169	60.07663		27.86069	15.32786	21.54316	26.40525	20.06583	28.18786
Repeat2	49.4245	54.96778	65.0804	63.60718	59.90654	64.00634		32.19458	13.18458	31.44682	21.19371	25.98292	24.54855
Repeat3	54.39316	53.32893	51.75794	57.29839	55.16569	42.53342		22.87472	9.455078	22.29456	28.30271	18.0063	18.17538

Fig2A: TDP2 invitro assay

0h 1h 3h 5h 7h 9h blank

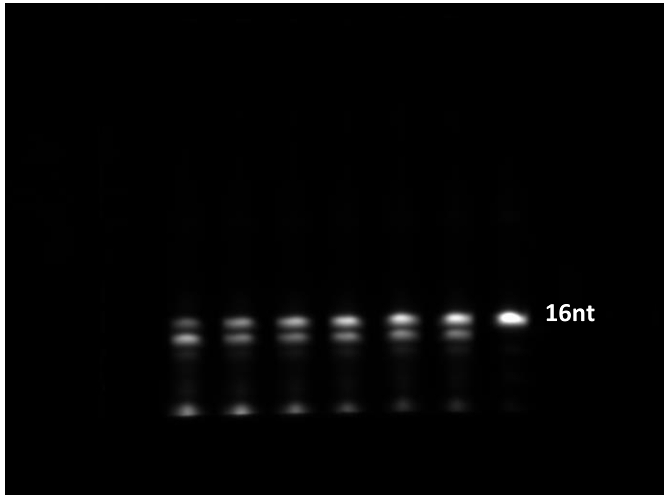

blank 0h 1h 3h 5h 7h 9h

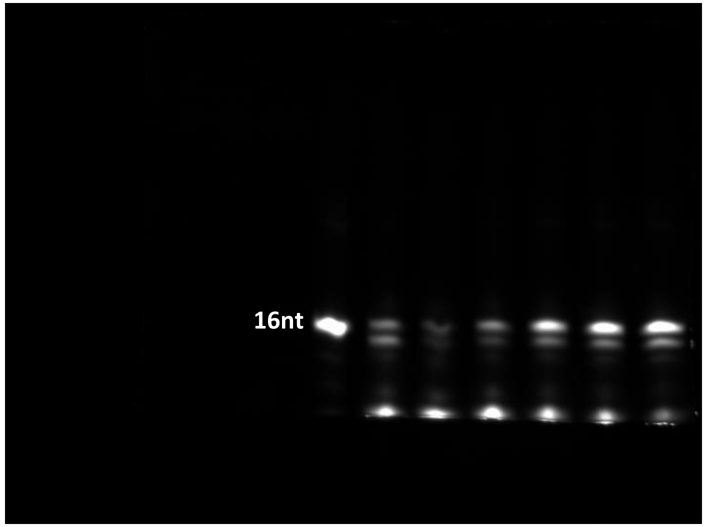

0h 1h 3h 5h 7h 9h blank

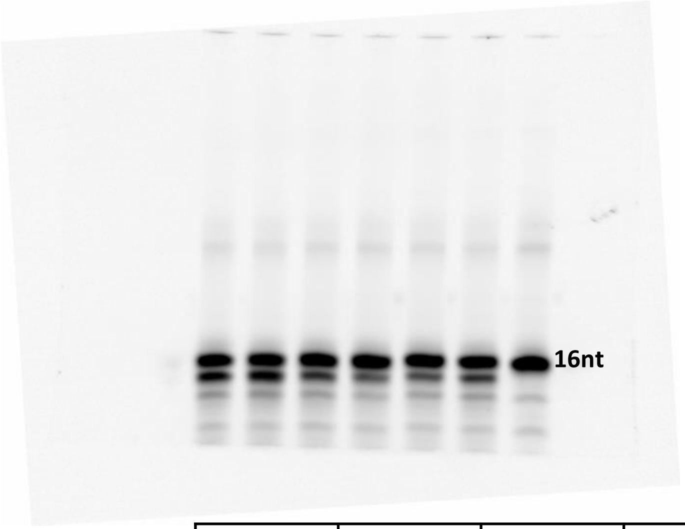

blank 0h1h 3h 5h7h

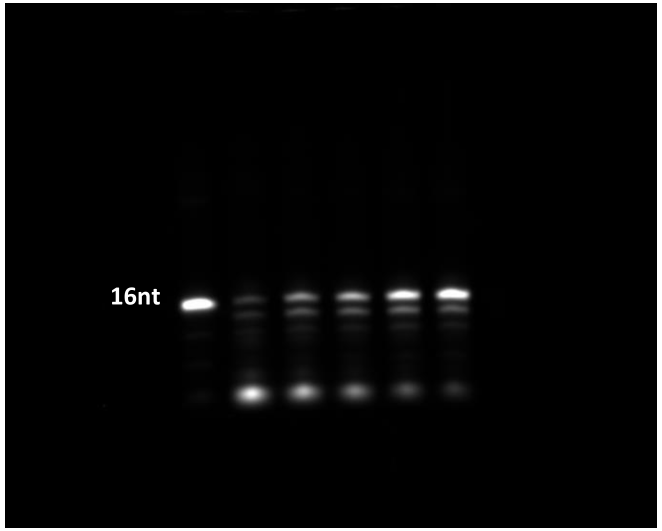

**%Cleavage**	**0h**	**1h**	**3h**	**5h**	**7h**	**9h**
Repeat1	57	42	34.46452	33.3543	32.72638	28.22608
Repeat2	52	40.5	22.74542	20	18.02505	20.71826
Repeat3	41	43	19	11.5	11	13
Repeat4	50	41	40	33	25	

Fig2B: TDP2 Western

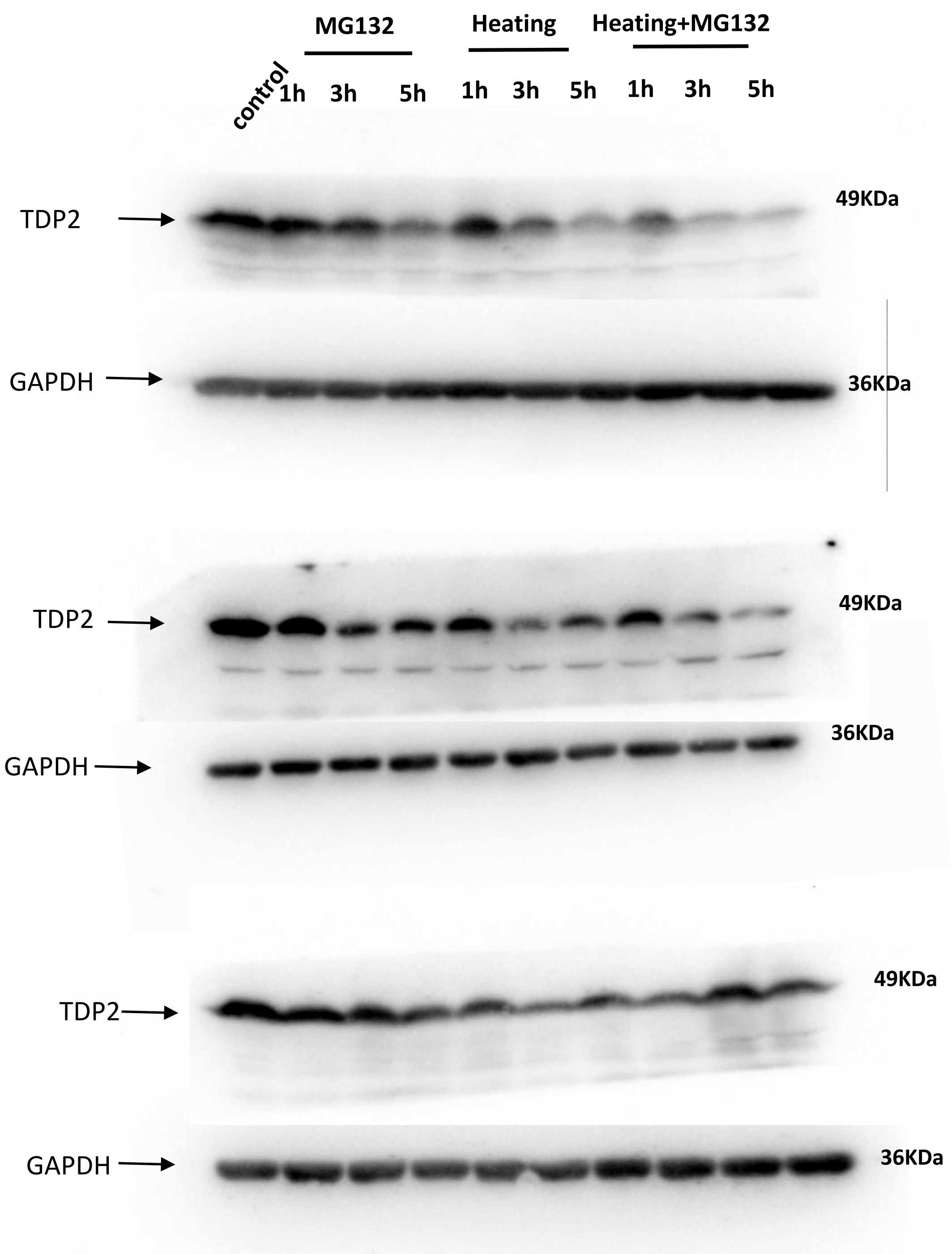

%Normalized volume intensity			
	Repeat1	Repeat2	Repeat3
control	100	100	100
MG132 1h	88.85992	53.86642	66.70549
MG132 3h	61.70755	21.00316	73.00844
MG132 5h	39.96519	24.76178	56.43512
heating 1h	61.96806	33.88544	56.71766
heating 3h	43.70052	11.37746	41.10401
heating 5h	21.18826	20.77166	43.0932
MG132 + heating 1h	38.01299	27.62734	34.58984
MG132 + heating 3h	16.71789	15.09612	54.34561
MG132 + heating 5h	17.13387	7.130026	34.87407

Fig2C: TDP2 invitro assay

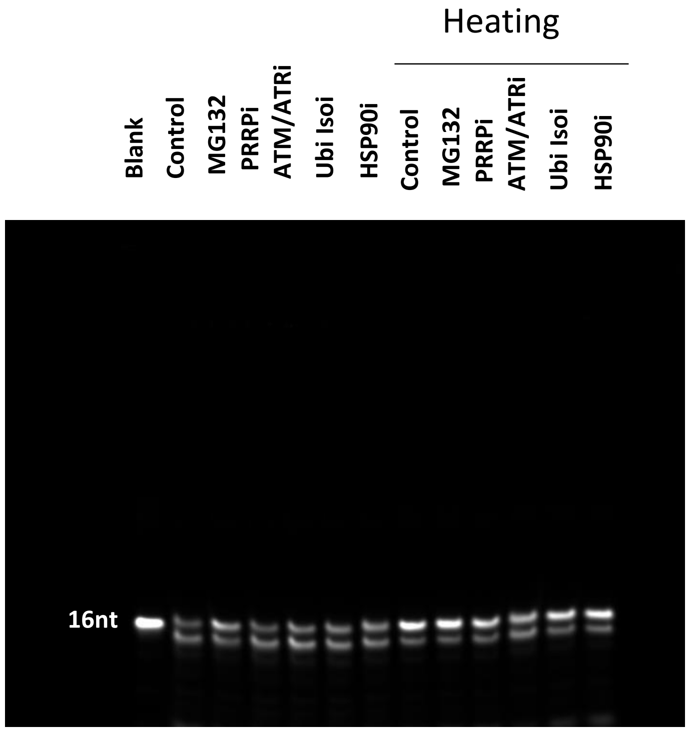

Repeat1

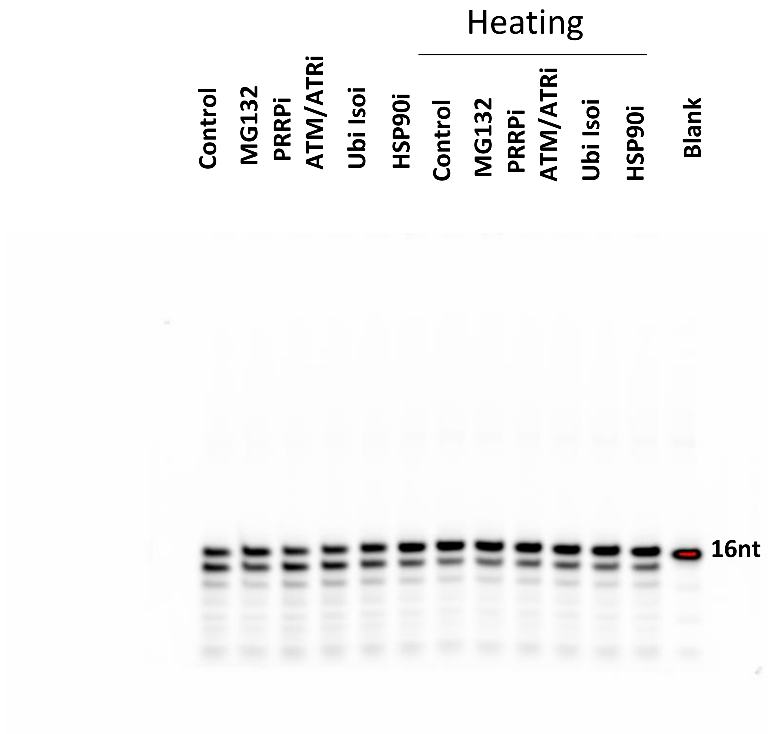

Repeat2

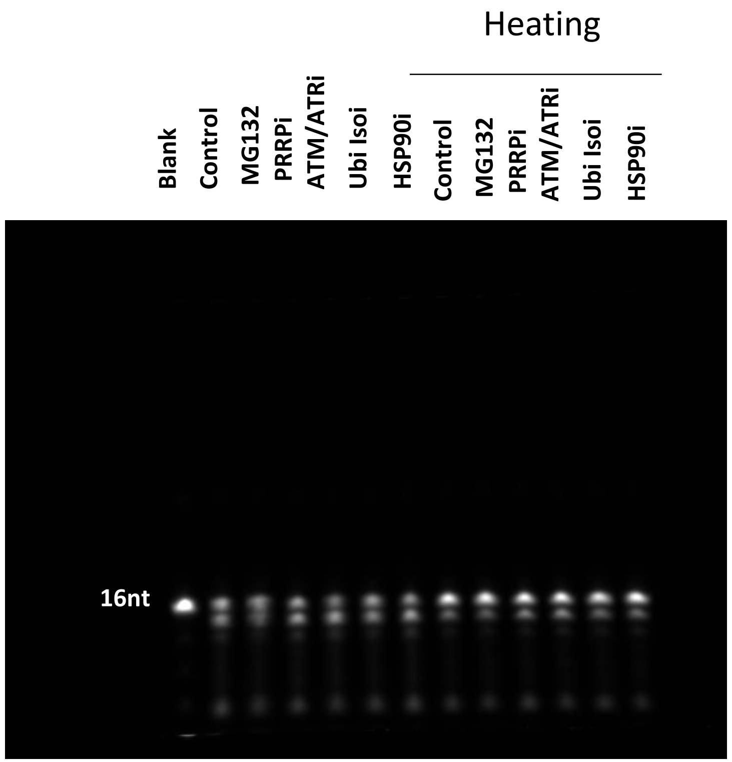

Repeat3

Fig2C: TDP2 invitro assay, continue

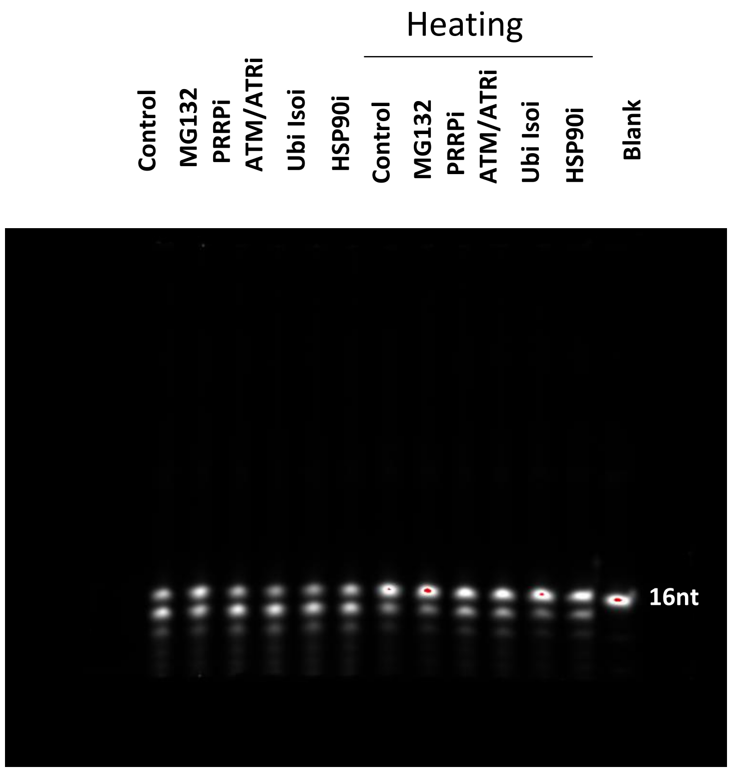

Repeat4

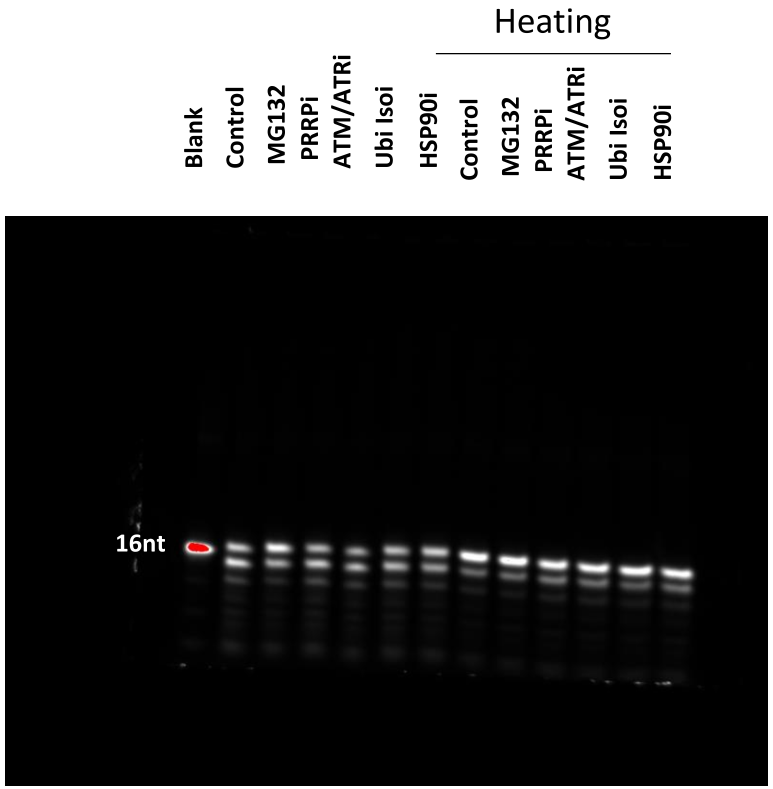

Repeat5

Fig2C: TDP2 invitro assay, continue

%Cleveage	37							43					
	Control	Proteasomei	PARP1i	Atm/Atri	Ubi Isoi	HSP90i		Control	Proteasomei	PARP1i	Atm/Atri	Ubi Isoi	HSP90i
Repeat1	57.12542	39.11175	57.55021	54.20614	49.03107	44.40487		26.06424	24.11538	30.41009	41.88216	31.08516	29.80286
Repeat2	52.0935	42.14672	57.42222	61.76515	60.19656	52.10208		25.17908	20.08203	33.86115	30.64224	17.8532	25.84065
Repeat3	52.92242	38.52909	53.8455	52.93266	49.32194	41.39175		23.0625	23.17492	29.04115	27.68463	22.50063	28.72457
Repeat4	46.06944	37.97119	49.4824	47.07979	38.62291	27.77775		23.75547	25.92907	26.14825	27.98453	25.54146	24.99295
Repeat5	42.2169	37.19907	48.71686	58.1911	43.68882	52.7925		24.73015	21.7516	29.18949	31.85882	26.63635	24.55925

Fig4 C: TDP2 invitro assay

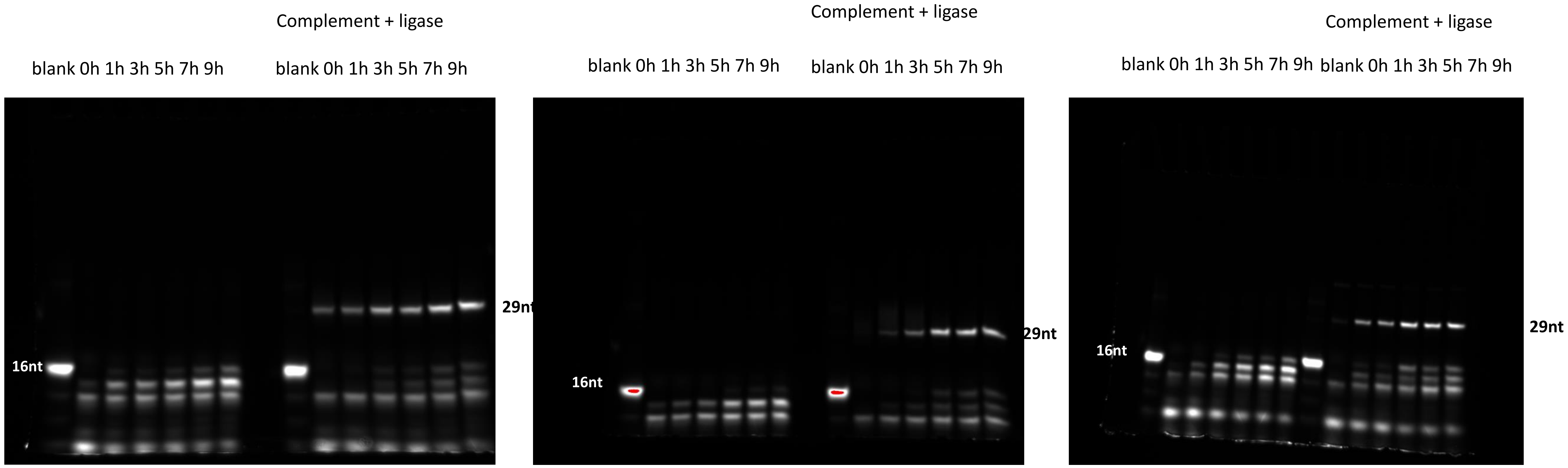

29nt substrate level	oh	1h	3h	5h	7h	9h
Repeat1	1	7.279684	8.477099	15.31179	14.09294	14.57037
Repeat2	1	5.22	7.753028	17.4405	17.83362	17.97073
Repeat3	1	4.235	6.351	12.112	12.5	13.388

Fig5 A: TDP1 invitro assay

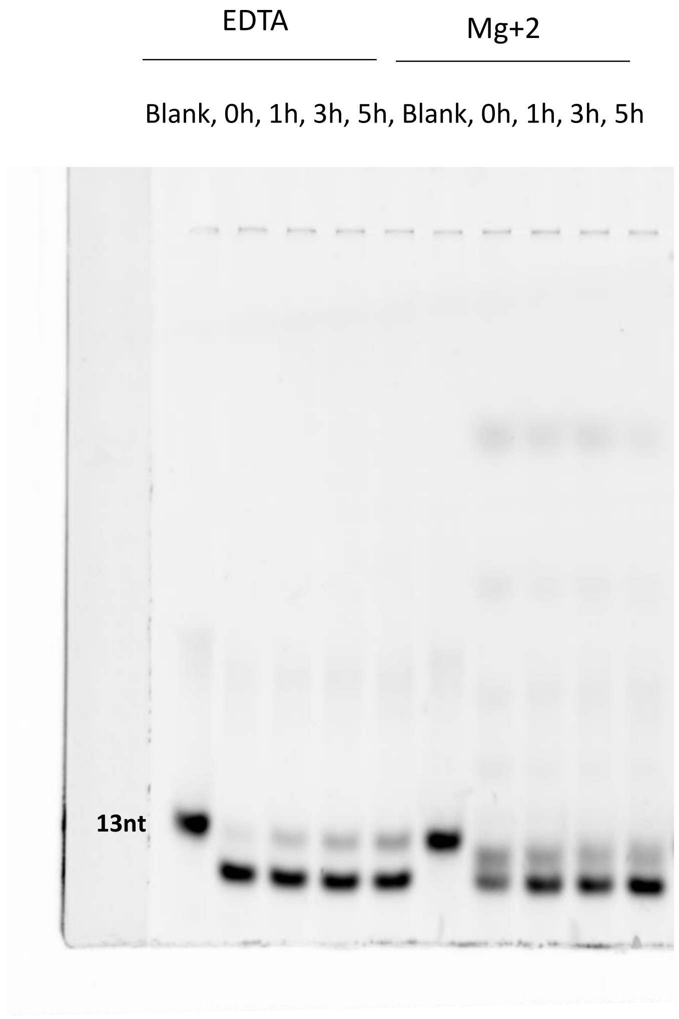

Repeat1

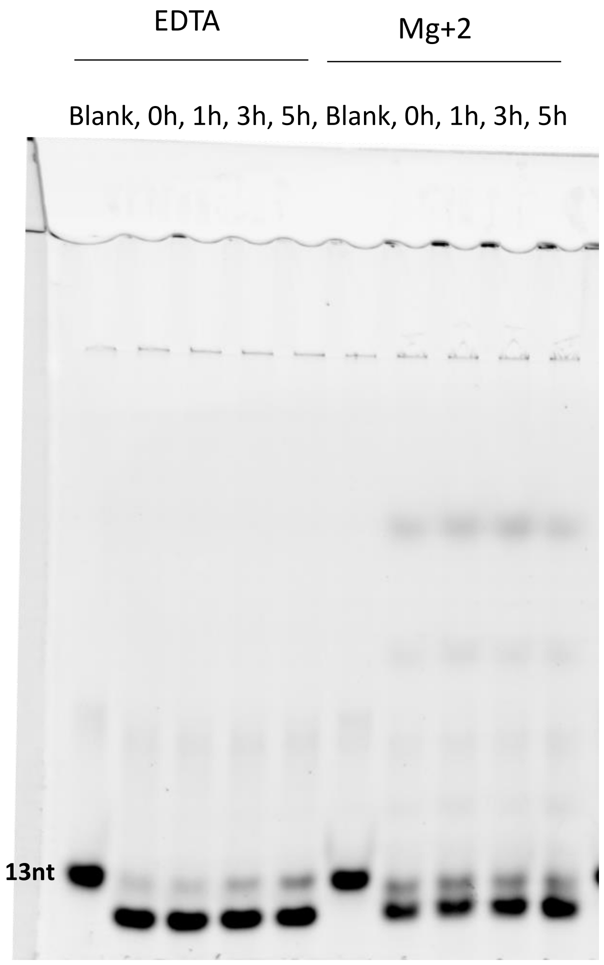

Repeat2

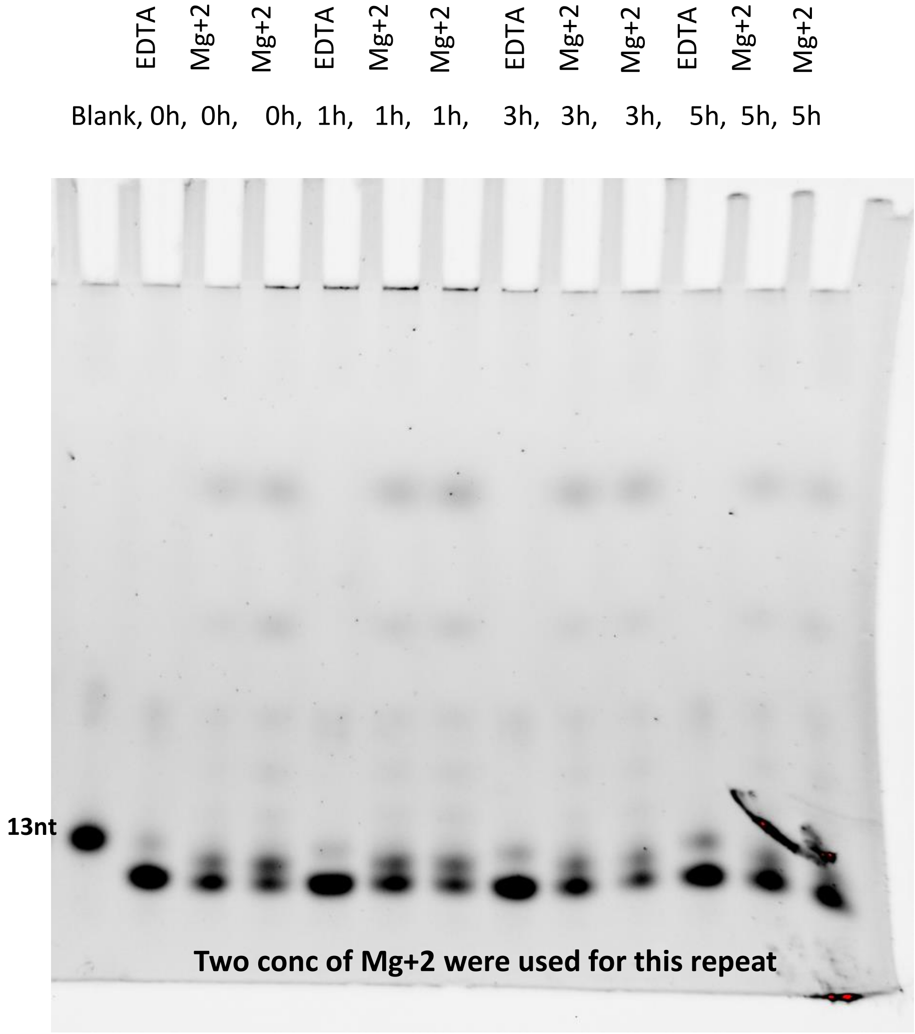

Repeat3

Fig5 A: TDP1 invitro assay, continue

		Repeat1	Repeat2	Repeat3
EDTA	0h	100	100	100
	1h	95.03922	97.66573	100.201
	3h	94.70588	97.01214	107.7387
	5h	95.98039	93.81046	109.5477
Mg+2	0h	30.39216	28.38469	21.00503
	1h	54.01961	47.43231	35.9799
	3h	59.21569	60.69374	45.91457
	5h	75	82.81979	79.19598

Fig5 C: TDP1 invitro assay

EDTA

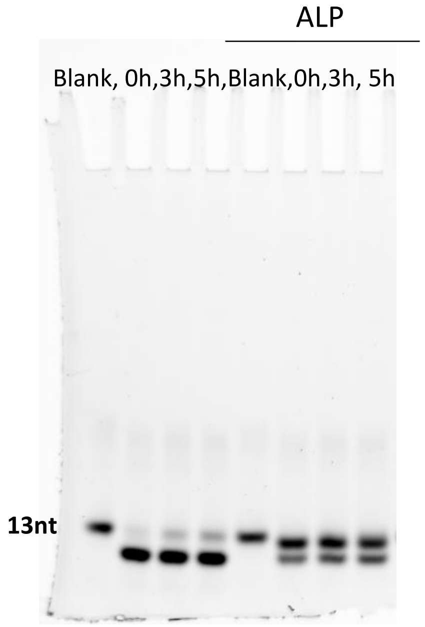

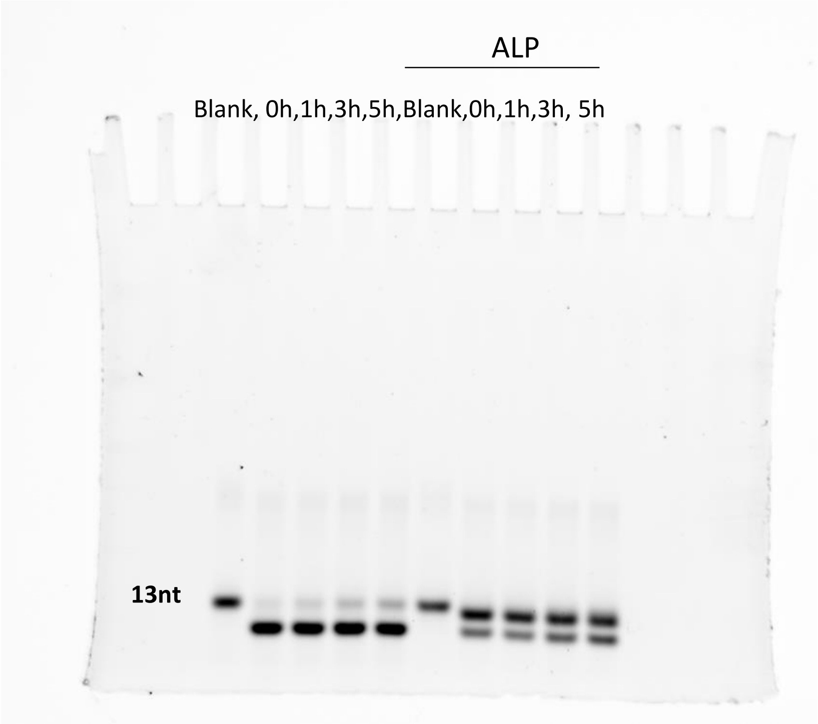

Mg+2

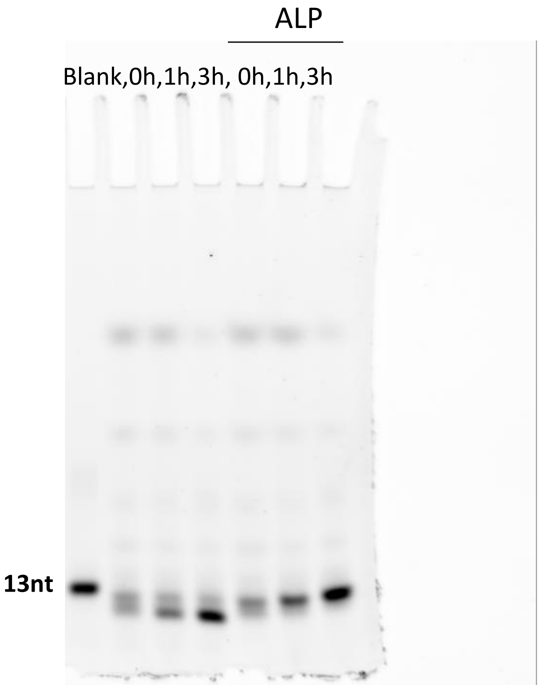

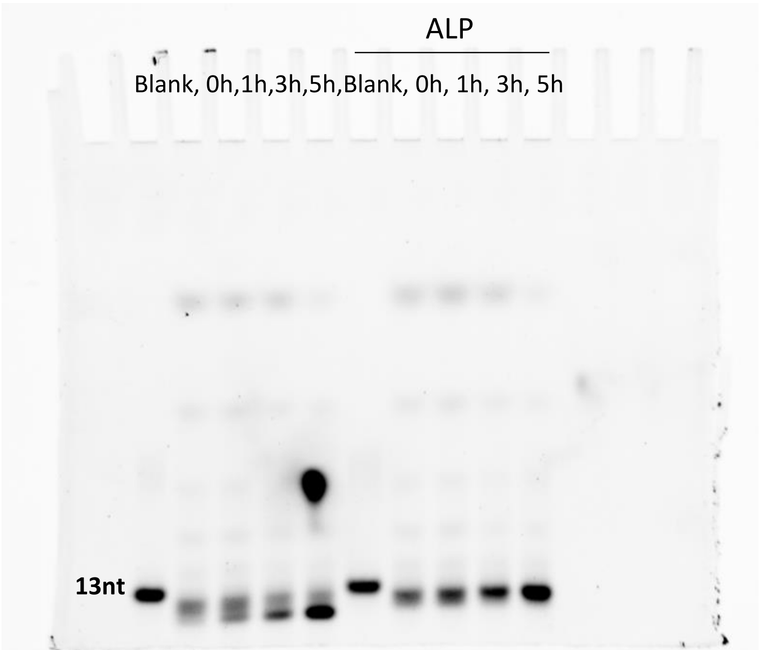

Fig5 E: TDP1 invitro

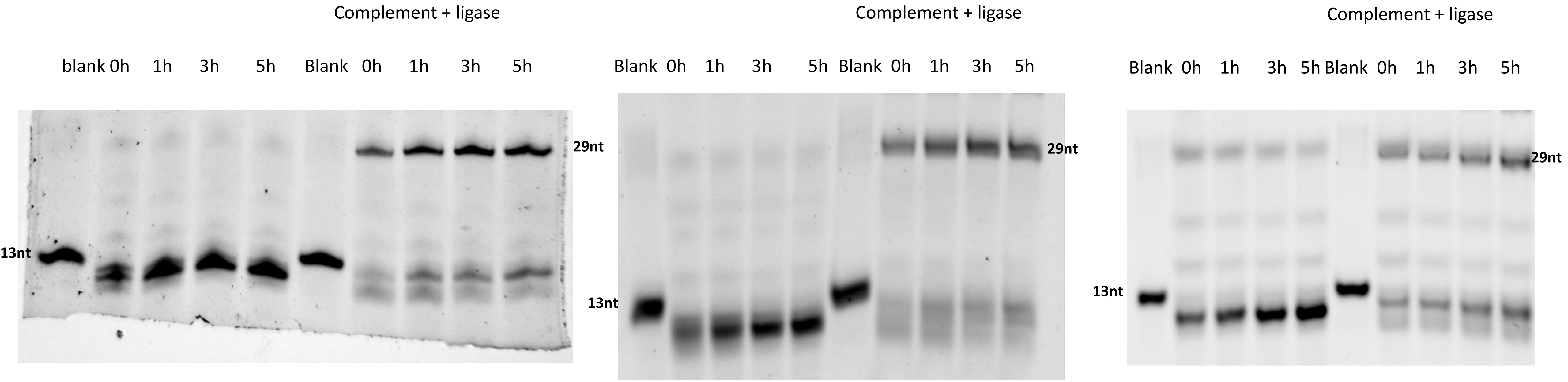

29nt substrate level	oh	1h	3h	5h
Repeat1	1	1.62	1.98	2.06
Repeat2	1	1.5	1.69	1.55
Repeat3	1	1.4	1.63	1.85

Sup Fig1A: TDP1 invitro

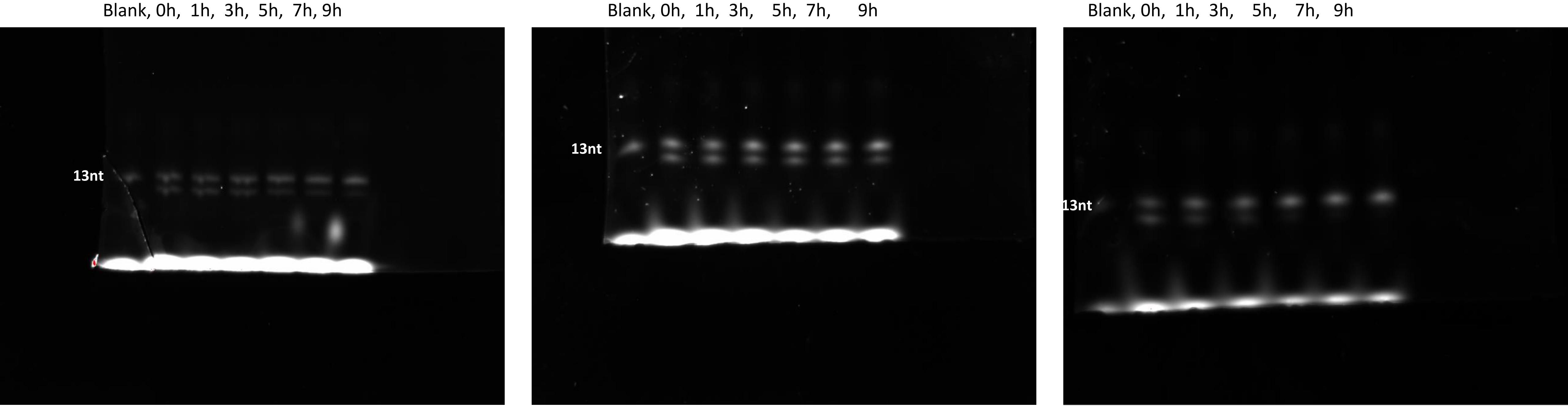

	0h	1h	3h	5h	7h	9h
Repeat1	51.20768	47.13295	45.29554	37.37342	36.75391	32.17669
Repeat2	48.86694	44.15135	36.21256	35.9338	36.33237	27.12692
Repeat3	51.14233	38.98247	29.61954	17.99809	17.23016	3.805741

Sup Fig1B: TDP1 invitro

No Recovery

Recovery

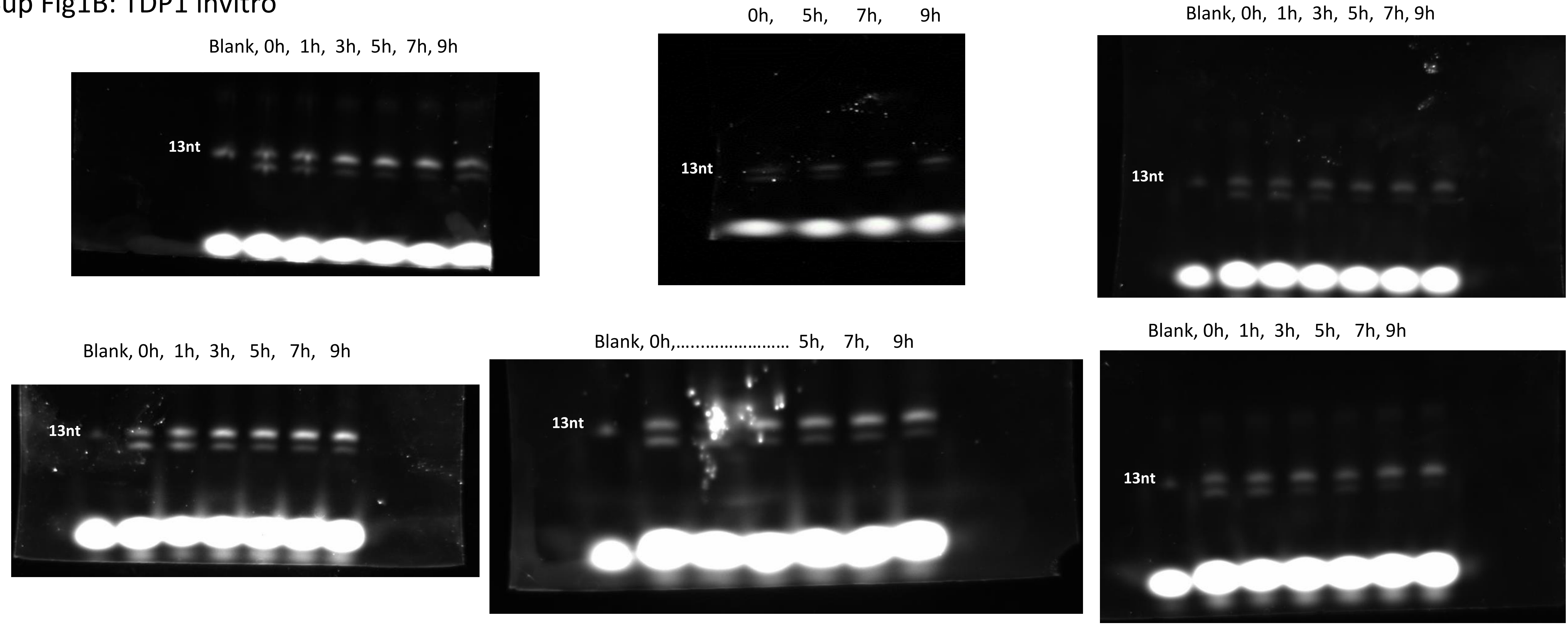

	no recovery				10h recovery		
0h	50.82744	45.55253	47.92951		47.91522	40.86479	44.7725
1h	29.42339		49.6724		37.86118		39.07691
3h	12.77287		31.44191		23.57195		30.10609
5h	9.856818	15.83982	27.57348		20.83397	19.98389	35.02694
7h	7.131401	13.06271	20.10335		14.56973	16.9328	28.20941
9h	19.52427	12.47891	23.11802		16.39244	13.59163	22.31429

Sup Fig1D: TDP1 invitro

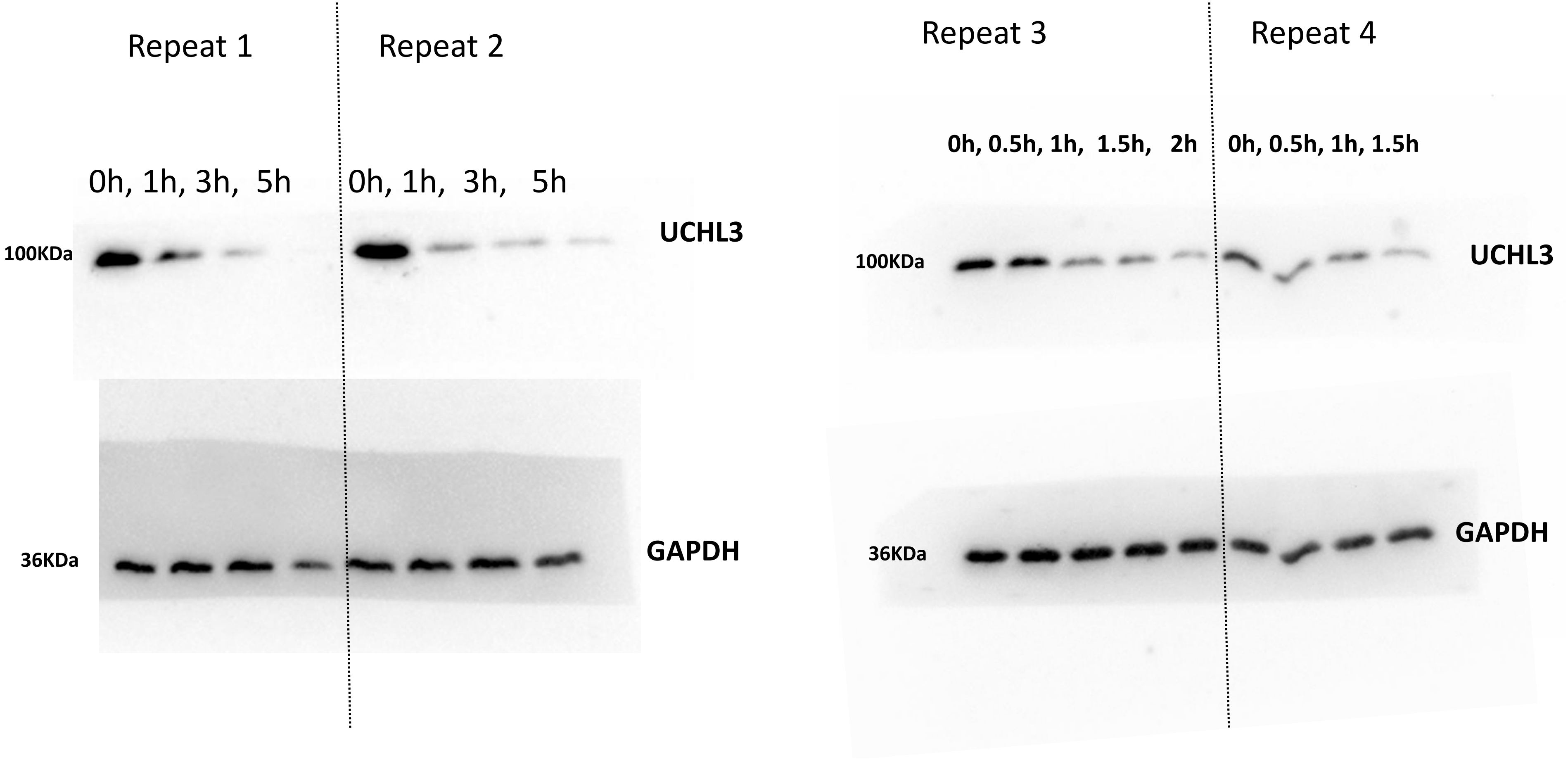

	0h	0.5	1h	1.5h
repeat1	100		29.92666	
repeat2	100		16.17816	
repeat3	100	77.60092	37.63786	33.27689
repeat4	100	67.75622	67.20804	33.99389

Sup Fig1E: TDP1 invitro

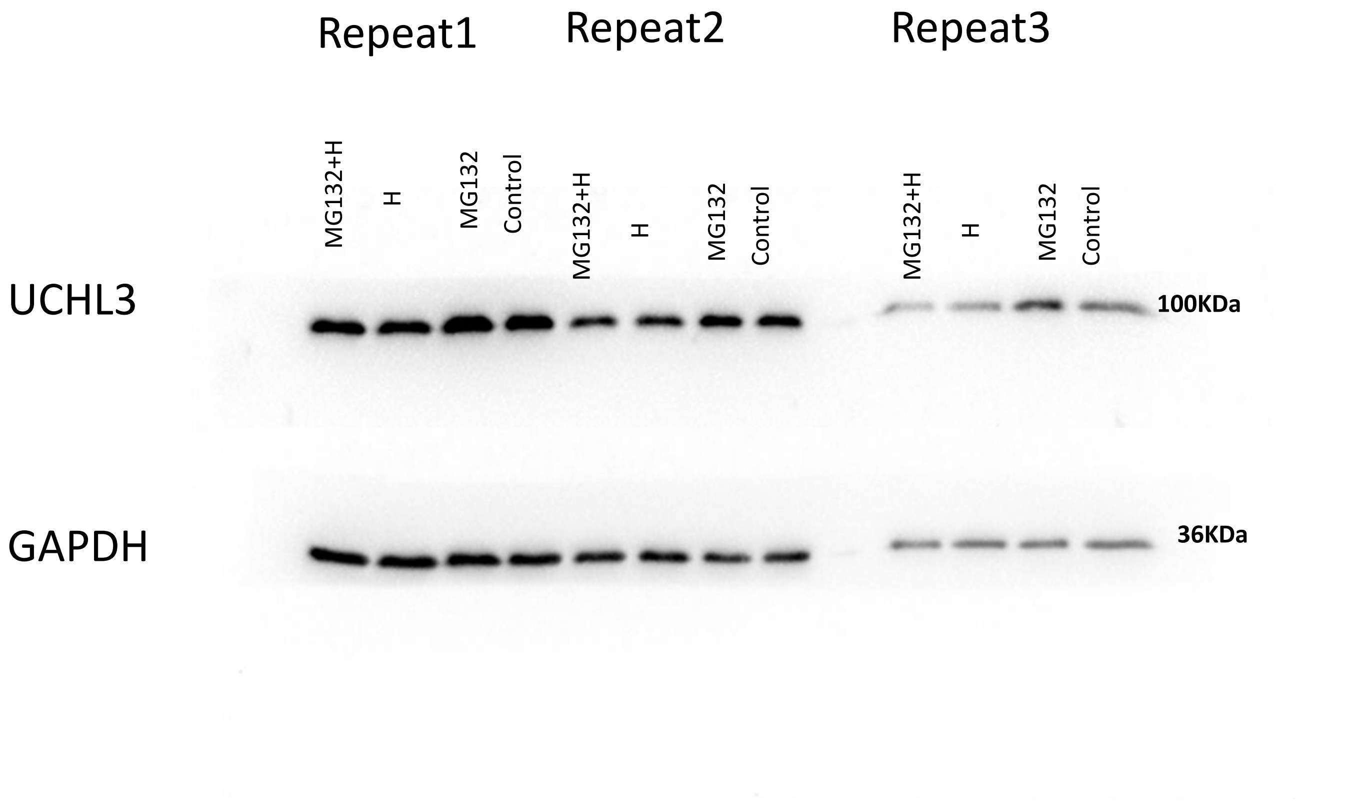

	Repeat1	Repeat2	Repeat3
MG132+H	82.57193	61.37013	42.90908
H	77.9621	66.22433	52.87964
MG132	106.2084	112.4492	129.7709
Control	100	100	100
